# Myxoma Virus Protein M029 Is a Dual Function Immunomodulator that Inhibits PKR and Also Conscripts RHA/DHX9 to Promote Expanded Host Tropism and Viral Replication

**DOI:** 10.1371/journal.ppat.1003465

**Published:** 2013-07-04

**Authors:** Masmudur M. Rahman, Jia Liu, Winnie M. Chan, Stefan Rothenburg, Grant McFadden

**Affiliations:** 1 Department of Molecular Genetics and Microbiology, University of Florida, Gainesville, Florida, United States of America; 2 Laboratory for Host-Specific Virology, Division of Biology, Kansas State University, Manhattan, Kansas, United States of America; University of Alberta, Canada

## Abstract

Myxoma virus (MYXV)-encoded protein M029 is a member of the poxvirus E3 family of dsRNA-binding proteins that antagonize the cellular interferon signaling pathways. In order to investigate additional functions of M029, we have constructed a series of targeted M029-minus (vMyx-M029KO and vMyx-M029ID) and V5-tagged M029 MYXV. We found that M029 plays a pivotal role in determining the cellular tropism of MYXV in all mammalian cells tested. The M029-minus viruses were able to replicate only in engineered cell lines that stably express a complementing protein, such as vaccinia E3, but underwent abortive or abated infection in all other tested mammalian cell lines. The M029-minus viruses were dramatically attenuated in susceptible host European rabbits and caused no observable signs of myxomatosis. Using V5-tagged M029 virus, we observed that M029 expressed as an early viral protein is localized in both the nuclear and cytosolic compartments in virus-infected cells, and is also incorporated into virions. Using proteomic approaches, we have identified Protein Kinase R (PKR) and RNA helicase A (RHA)/DHX9 as two cellular binding partners of M029 protein. In virus-infected cells, M029 interacts with PKR in a dsRNA-dependent manner, while binding with DHX9 was not dependent on dsRNA. Significantly, PKR knockdown in human cells rescued the replication defect of the M029-knockout viruses. Unexpectedly, this rescue of M029-minus virus replication by PKR depletion could then be reversed by RHA/DHX9 knockdown in human monocytic THP1 cells. This indicates that M029 not only inhibits generic PKR anti-viral pathways, but also binds and conscripts RHA/DHX9 as a pro-viral effector to promote virus replication in THP1 cells. Thus, M029 is a critical host range and virulence factor for MYXV that is required for replication in all mammalian cells by antagonizing PKR-mediated anti-viral functions, and also conscripts pro-viral RHA/DHX9 to promote viral replication specifically in myeloid cells.

## Author Summary

Poxviruses exploit diverse strategies to modulate host anti-viral responses in order to achieve broad cellular tropism and replication. Here we report the findings that Myxoma virus (MYXV), a rabbit-specific poxvirus, expresses a viral protein M029 that possesses dual immunomodulatory functions. M029 binds and inhibits the anti-viral functions of protein kinase R (PKR) and also binds and conscripts the pro-viral activities of another cellular protein, RNA helicase A (RHA/DHX9), a member of the DEXD/H box family of proteins. Engineered M029-minus MYXVs did not cause lethal disease myxomatosis in the European rabbits. M029-minus MYXVs were also unable to replicate in diverse mammalian cell types, but can be rescued by knocking down the expression of PKR. However, this rescue of M029-minus virus replication could then be reversed by RHA/DHX9 knockdown in human myeloid cells. These findings reveal a novel strategy used by a single viral immunomodulatory protein that both inhibits a host anti-viral factor and additionally conscripting a host pro-viral factor to expand viral tropism in a wider range of target mammalian cells.

## Introduction

Poxviruses encode dozens of modulatory proteins that are involved in evasion of host immune responses and are critical virulence factors needed for viral pathogenesis [1], [2], [3]. The poxvirus E3 family of immune evasion proteins antagonizes multiple anti-viral cellular signaling pathways that are primarily induced by interferon (IFN). The two best-characterized cellular targets of the E3 protein, encoded by the vaccinia virus (VACV) *E3L* gene, are PKR and 2′-5′-oligoadenylate synthetase (2′-5′OAS), both of which are activated by dsRNA and trigger a global inhibition of protein synthesis to combat progression of the viral replication cycle [4], [5], [6], [7]. VACV E3 also blocks the activation of IFN regulatory transcription factors 3 (IRF-3) and 7 (IRF-7) [8], nuclear factor κB (NF-κB) [9], [10], and IFN-stimulated gene 15 (ISG15) [11], all of which contribute to the anti-viral state of IFN-treated cells. This subversion of host anti-viral functions by VACV E3 is primarily mediated by at least two protein domains: a carboxy (C)-terminal dsRNA-binding domain and an amino (N)-terminal Z-DNA binding domain [12], [13], [14], [15], both of which are required for full virus virulence in mice.

Targeted deletion of the *E3L* gene of VACV results in reduced cellular tropism in certain cultured cell lines and increased sensitivity to the inhibitory effects of IFNs [16]. However, the *in vitro* and *in vivo* roles of the two N- and C- terminal domains of E3 differ significantly, for reasons not yet clearly defined. For example, the N-terminal domain of E3 is not required for VACV replication in at least some cell types, but is required for *in vivo* pathogenicity [13], [14], [17]. In addition, the N–terminal domain is required for inhibition of the type I IFN response in mice and in mouse embryo fibroblasts (MEFs) [15]. More recent studies suggest that E3 also plays a role in antagonizing the cellular sensing pathways activated by dsDNA and dsRNAs as mediated by various cellular PRRs [18], [19], [20].

Several studies indicate that E3 function(s) in VACV can be complemented *in vitro*, but not *in vivo*, by various related viral and bacterial proteins with dsRNA-binding capacity. For example, influenza virus NS1, reovirus S4, *Escherichia coli* RNase III or the Orf Virus E3 homologue could complement some E3 host range functions in cultured cells but could not restore pathogenicity of E3L-minus VACV [21], [22], [23], [24]. This suggests that the dsRNA binding properties of these proteins alone may not be sufficient for rescuing VACV *in vivo* replication and virulence in the absence of E3. Moreover, E3 orthologs derived from poxviruses of other genera were also not able to restore full VACV pathogenicity in the absence of E3 [25]. These various tested E3 orthologs were also significantly diverged in terms of their capacity to complement the host range functions of E3 in cultured mammalian cells. These results all suggest that the various poxvirus E3 orthologs might have acquired unique host immune modulatory functions and have different cellular target(s), depending on the evolutionary histories of the specific virus. In other words, the dsRNA-binding property alone may not be sufficient to explain all of the many known E3 functions.

Myxoma virus (MYXV) is a rabbit-specific Leporipoxvirus that encodes a portfolio of immunomodulatory proteins, many of which are very distinct from those encoded by the Orthopoxvirus VACV [26]. Of the host-interactive modulatory proteins that are relatively more similar between the two viruses, the MYXV M029 is a truncated ortholog of VACV E3 that lacks a significant portion of N-terminal Z-DNA binding domain of VACV E3 [26]. Replacement of E3 with M029 in the VACV background can restore some of the host range functions of E3 (for example, in HeLa cells) presumably by suppression of common cellular anti-viral activities [25]. However, M029 replacement was not able to restore pathogenicity of E3-deficient VACV *in vivo*. This suggests that, M029 likely also has distinct roles in terms of MYXV biology, such as virus pathogenicity in rabbits, anti-viral activity and/or host range functions.

Here we report several important and novel roles for M029 in MYXV tropism and pathogenicity, in terms of both extending viral host range in cultured mammalian cells, as well as acting as a virulence factor for myxomatosis in European rabbits. When M029 expression was abrogated, the M029-minus MYXV constructs were defective for replication in essentially all mammalian cell types tested and failed to cause any aspects of myxomatosis disease in rabbits. We exploited a proteomic approach to reveal that M029 is a major host range determinant for MYXV, with at least two distinct biological functions. One is to bind PKR protein in a dsRNA-dependent fashion in order to antagonize PKR anti-viral responses in a wide variety of mammalian cell lineages from multiple species. M029 also interacts with a cellular member of the DEXD/H box family of proteins, RHA/DHX9, in a dsRNA-independent fashion. However, in contrast to PKR, instead of inhibiting this second target, M029 binds and conscripts RHA/DHX9 to promote MYXV replication in a cell type dependent manner. Thus, these two distinct cellular protein interactions of M029 represent the first example of a single viral immunomodulatory protein interacting with two distinct host binding partners, one of which (PKR) is inhibited to alleviate anti-viral responses and the other (RHA/DHX9) is redeployed as a novel pro-viral effector to expand viral tropism in a select subset of target mammalian cells.

## Results

### M029 is a critical host range factor for MYXV in rabbit cells

**Figure 1:**
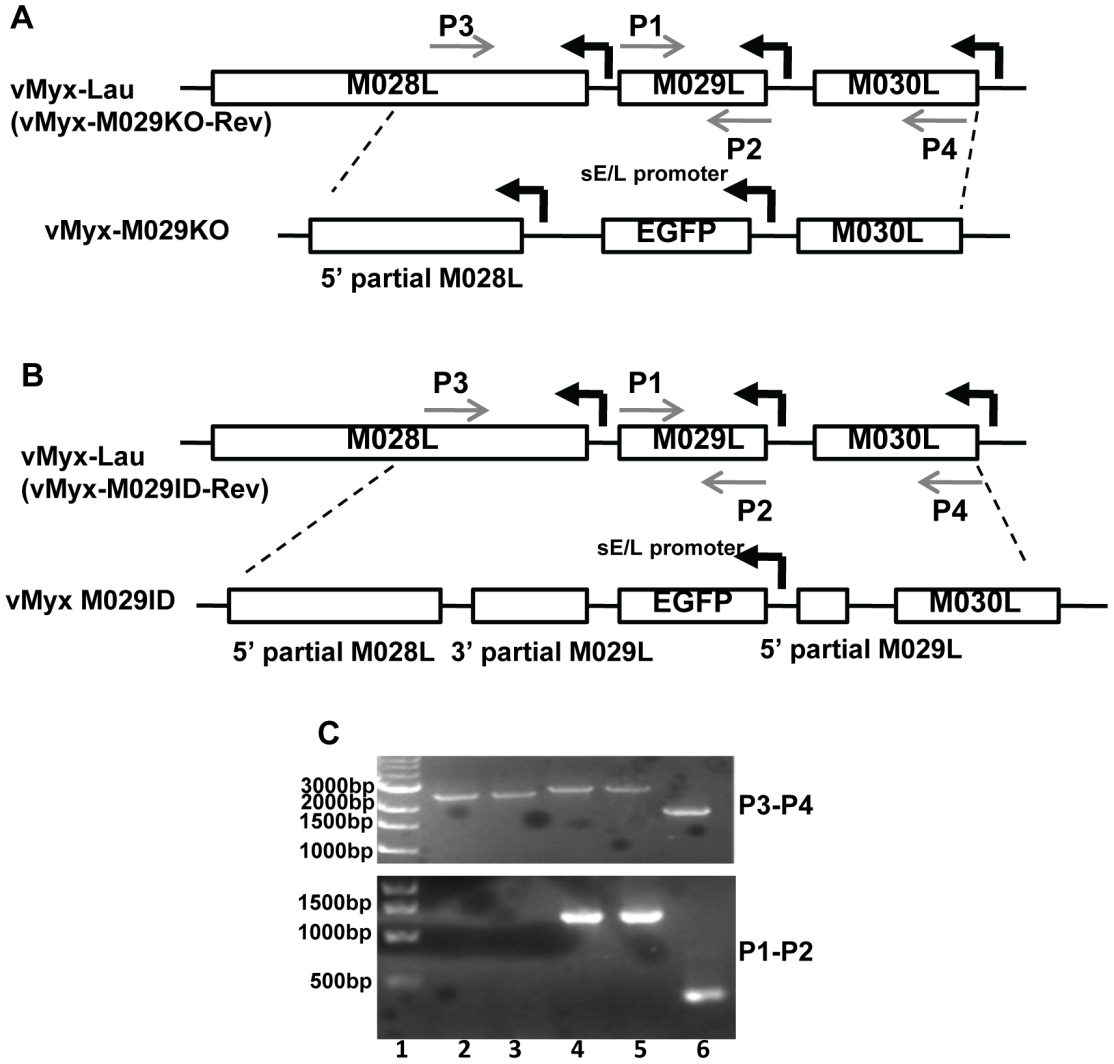
Construction of recombinant MYXVs. A) Construction of vMyx-M029KO virus. The M029 coding sequence was replaced by a DNA cassette where eGFP is expressed under a poxvirus synthetic early/late promoter (sE/L). The final recombination plasmid has 5′ partial M028L sequence, eGFP and M030L sequence and was constructed by three-element recombination of the Multisite Gateway system. The revertant virus vMyx-M029KO-Rev was constructed by replacing the eGFP cassette in the knockout virus with a plasmid having M028-M029-M030 gene sequence. B) Construction of vMyx-M029ID virus. A DNA cassette where eGFP expressed under a poxvirus synthetic early/late promoter (sE/L) was inserted within M029 gene for disruption of M029 ORF. The final recombination plasmid has 5′ partial M028L sequence, 5′ partial M029L sequence, eGFP, 3′ partial M029L sequence and M030L sequence and was constructed by three-element recombination of the Multisite Gateway system. The revertant virus vMyx-M029ID-Rev was constructed by replacing the eGFP cassette with a plasmid having the intact M028-M029-M030 gene sequence. C) Confirmation of purified vMyx-M029KO and vMyx-M029ID viruses. PCR using primers combination of 3 and 4 (top panel) and 1 and 2 (bottom panel) used for viral DNA from vMyx-M029KO (lanes 2 and 3), vMyx-M029ID (lanes 4 and 5), vMyx-Lau (lane 6). Lane 1 represents known size DNA ladder.

**Figure 2:**
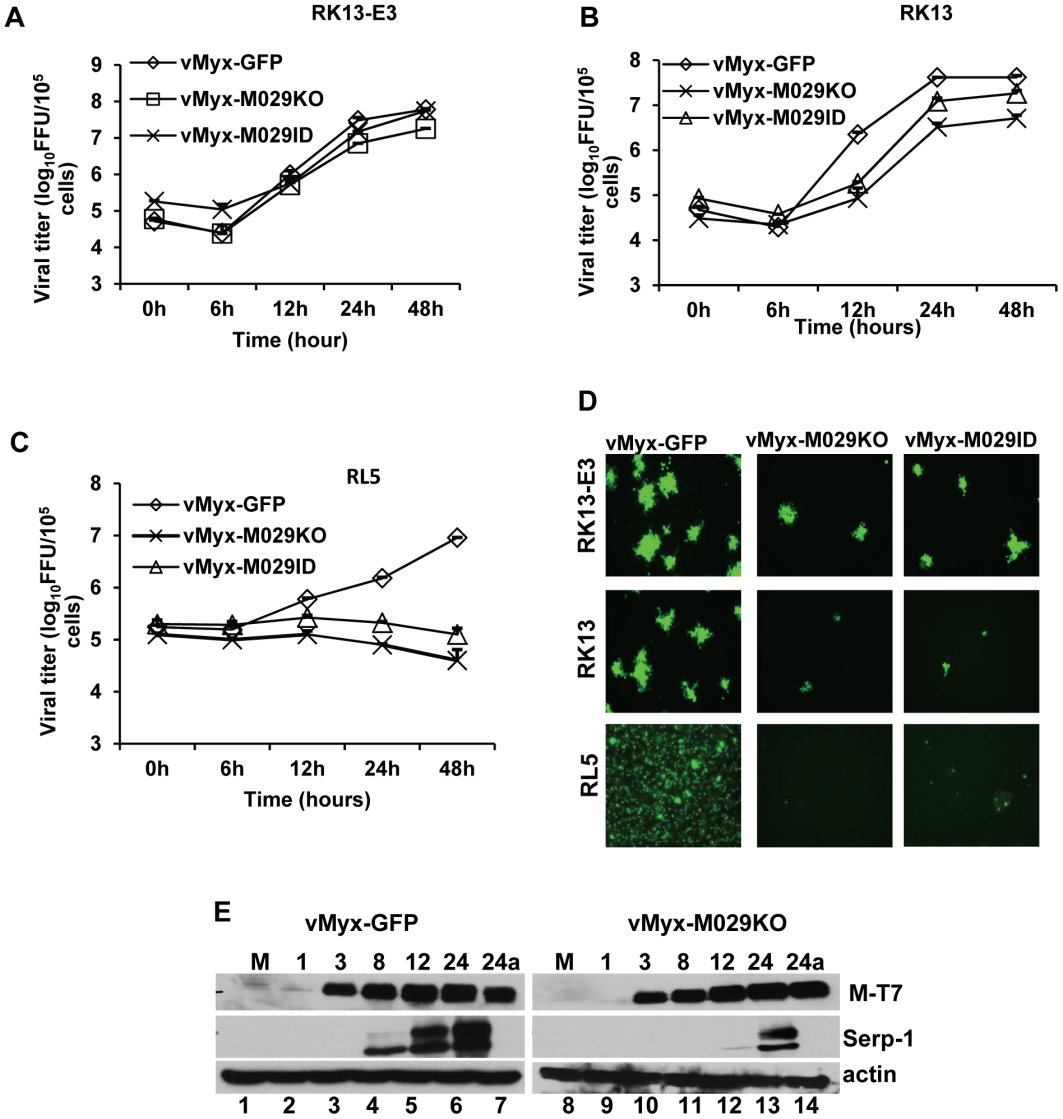
M029 is required for optimal MYXV replication in rabbit cells. Single step growth curves of MYXV infection in A) RK13-E3, B) RK13 and C) RL5 T lymphocytes. The indicated cells were infected with vMyx-GFP, vMyx-M029KO or vMyx-M029ID at an MOI of 5, and then cells were collected at 0, 6, 12, 24, and 48 h p.i. The virus titers were determined in triplicate following serial dilution onto RK13-E3 cells. Data are representative of two independent experiments. D) Fluorescence microscope images of infected RK13-E3 (top panels), RK13 (middle panels) and RL5 (bottom panels) cells. The images were taken using an inverted fluorescence microscope using a lens with ×10 magnification at 48 h p.i. The cells were infected at an MOI of 0.05 for RK13-E3 and RK13 and an MOI of 3 for RL5 with vMyx-GFP (left column), vMyx-M029KO (middle column) and vMyx-M029ID (right column). E) Expression of viral early (M-T7) and late (Serp-1) proteins in the infected RK13 cells. RK13 cells were left uninfected (M) (lanes 1 and 8) or were infected with vMyxGFP (lanes 2 to 7) or vMyx-M029KO (lanes 9 to 14) at an MOI of 3. Cells were collected at 1 (lanes 2 and 9), 3 (lanes 3 and 10), 8 (lanes 4 and 11), 12 (lanes 5 and 12), 24 (lanes 6 and 13) and 24a in the presence of AraC (lanes 7 and 14) h p.i. The membranes were first probed with anti-Serp-1 antibody, stripped and probed for M-T7 and actin (loading control).

MYXV M029 has a single predicted dsRNA-binding domain, which displays approximately 45% sequence identity with the C-terminal dsRNA-binding domain of VACV E3, but it lacks the predicted N-terminal Z DNA binding motif that is found in E3. In order to study the biological functions of M029, we have disrupted M029 ORF by either inserting an eGFP expression cassette driven by a poxvirus synthetic early/late promoter (pE/L) [27] within the ORF (vMyx-M029ID) or replacing the entire M029 ORF with the same eGFP cassette (vMyx-M029KO) (Fig. 1). However, although we were able to detect eGFP-expressing viruses in the crude infection/transfection mixtures used to construct these viruses, we were not able to isolate any pure M029-defective recombinant viruses (i.e. expressing eGFP) in rabbit RK13, primate BSC40, hamster BHK21 or any other human cell lines that are normally permissive for wildtype MYXV replication. Instead, cloned M029-minus viruses were able to form foci only in engineered RK13 cell lines that constitutively express VACV E3 (RK13-E3) alone or expressed both E3 and K3 (RK13-E3K3) from VACV. Using these complementing cell lines, we were able to purify both M029-defective MYXVs, vMyx-M029KO (i.e. M029 knockout) and vMyx-M029ID (i.e. M029 insertional disruption). Both vMyx-M029KO and vMyx-M029ID viruses replicated normally in RK13-E3 (Fig. 2A) and RK13-E3K3 (not shown) and yielded progeny titers similar to control wildtype MYXV, called vMyx-GFP (MYXV expressing an eGFP cassette located at an intergenic site between M135 and M136, and driven by a poxvirus synthetic E/L promoter) [27], [28] as assessed by single step growth curve. These M029-minus and M029-expressing viruses formed similarly sized foci in RK13-E3 cells (Fig. 2D). However, in the parental RK13 cell line both vMyx-M029KO and vMyx-M029ID viruses formed much smaller foci, indicating a defect in virus replication and/or spread (Fig. 2D). When analyzed by single step growth curve, the virus titers for the M029-defective viruses were consistently lower, with more than one log difference at 12 hpi when compared with vMyx-GFP (Fig. 2B). This difference was accompanied by delayed viral late protein synthesis (as detected by Serp-1 levels on Western blots) in RK13 cells from both M029 mutant viruses (Fig. 2E, middle panels). However, there was no difference in the synthesis or kinetics of viral early proteins as detected by measuring M-T7 expression (Fig. 2E, top panels). In contrast, in RL5, a rabbit CD4+ T cell line, infection with either vMyx-M029KO or vMyx-M029ID viruses were abortive compared to the control vMyx-GFP replication (Fig. 2C). No late protein synthesis at all was detected in RL5 cells after infection with either vMyx-M029KO or vMyx-M029ID viruses (not shown).

### M029 is essential for replication of MYXV in all cell lines tested that originate from human or other non-lagomorph sources

**Figure 3:**
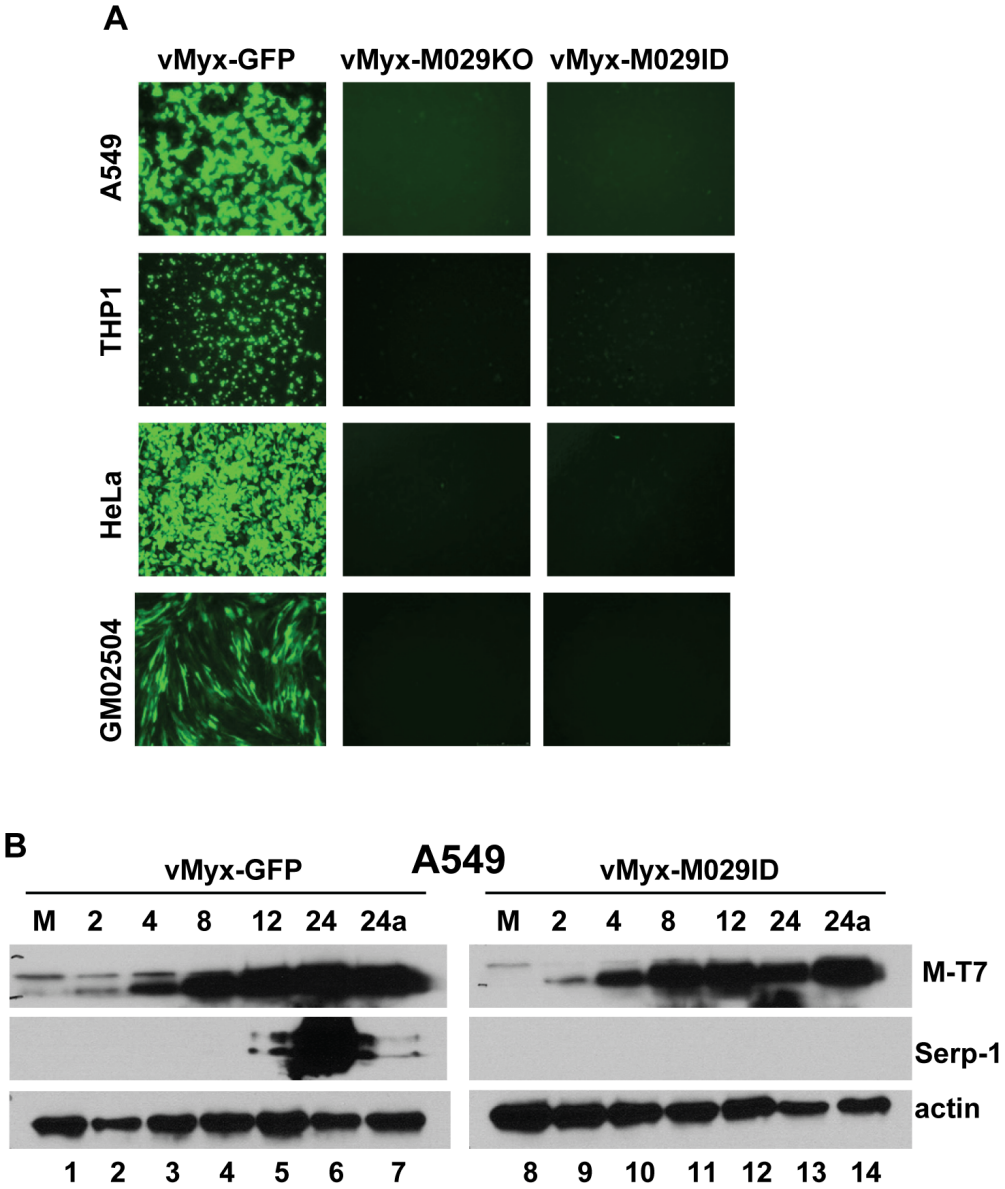
M029 is essential for MYXV replication in diverse human cells. A) Fluorescence microscope images of infected human cell lines A549 (top panels), THP1 (second panels), HeLa (third panels) and GM02504 (bottom panels). The images were taken using an inverted fluorescence microscope using a lens with ×10 magnification at 48 h p.i. The cells were infected with vMyx-GFP (left column), vMyx-M029KO (middle column) and vMyx-M029ID (right column). B) Expression of viral early (M-T7) and late (Serp-1) proteins in the infected A549 cells. A549 cells were left uninfected (M) (lanes 1 and 8) or were infected with vMyxGFP (lanes 2 to 7) or vMyx-M029ID (lanes 9 to 14) at an MOI of 3. Cells were collected at 2 (lanes 2 and 9), 4 (lanes 3 and 10), 8 (lanes 4 and 11), 12 (lanes 5 and 12), 24 (lanes 6 and 13) and 24a in the presence of AraC (lanes 7 and 14) h p.i. The membranes were first probed with anti-Serp-1 antibody, stripped and probed for M-T7 and actin (loading control).

**Figure 4:**
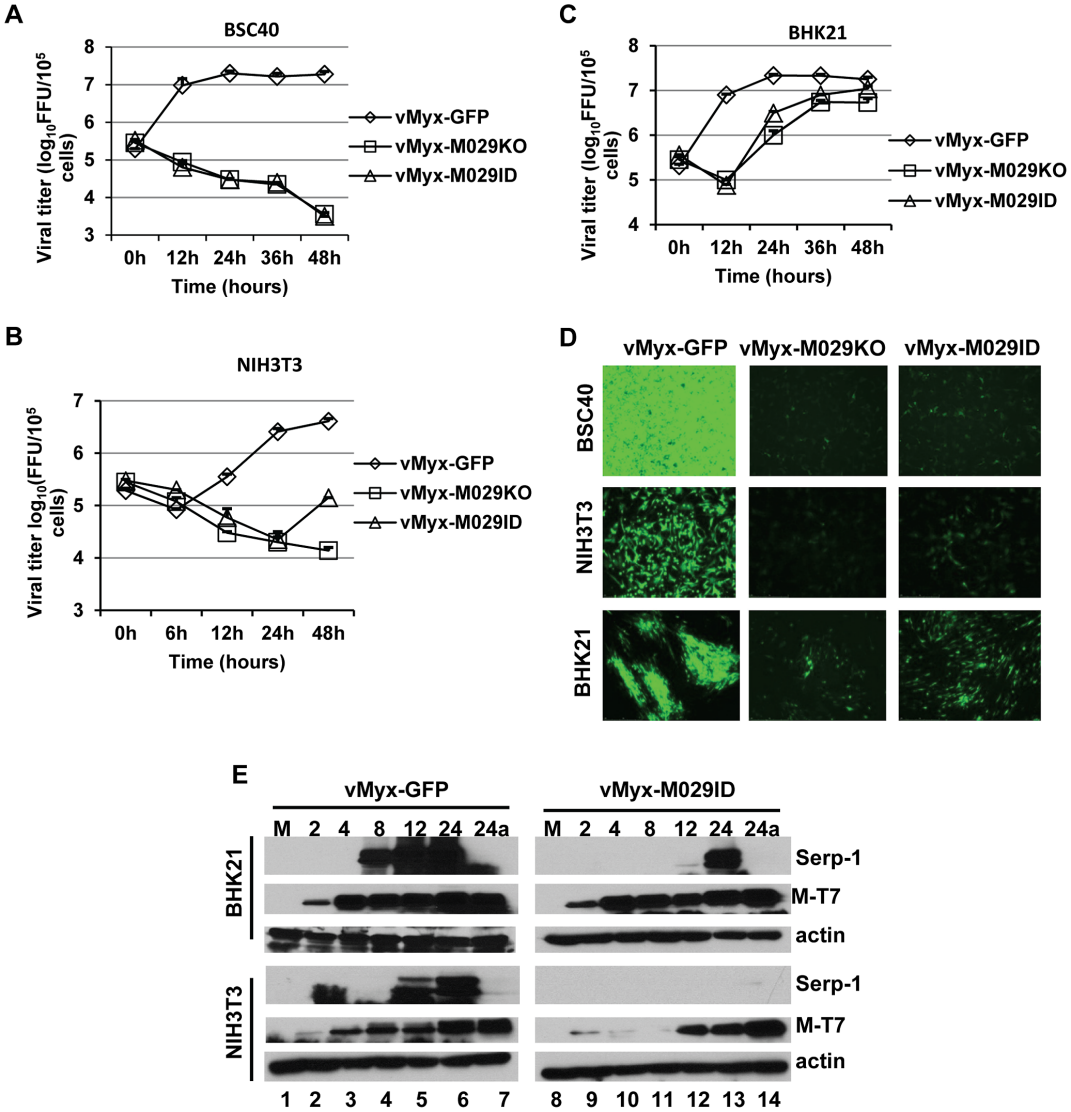
M029 is essential for MYXV replication in cell lines originated from diverse species. Single step growth curves of MYXV infection in A) BSC40, B) NIH3T3 and C) BHK21 cells. The indicated cells were infected with vMyx-GFP, vMyx-M029KO or vMyx-M029ID at an MOI of 5, and then cells were collected at 0, 6, 12, 24, 36, and 48 h p.i. virus titers were determined in triplicate following serial dilution onto RK13-E3 cells. Data are representative of two independent experiments. D) Fluorescence microscope images of infected BSC40 (top panels), NIH3T3 (middle panels) and BHK21 (bottom panels) cells. The images were taken using an inverted fluorescence microscope using a lens with ×10 magnification at 48 h p.i. The cells were infected with vMyx-GFP (left column), vMyx-M029KO (middle column) and vMyx-M029ID (right column). E) Expression of viral early (M-T7) and late (Serp-1) proteins in the infected BHK21 and NIH3T3 cells. BHK21 and NIH3T3 cells were left uninfected (M) (lanes 1 and 8) or were infected with vMyxGFP (lanes 2 to 7) or vMyx-M029ID (lanes 9 to 14) at an MOI of 3. Cells were collected at 2 (lanes 2 and 9), 4 (lanes 3 and 10), 8 (lanes 4 and 11), 12 (lanes 5 and 12), 24 (lanes 6 and 13) and 24a in the presence of AraC (lanes 7 and 14) h p.i. The membranes were first probed with anti-Serp-1 antibody, stripped and probed for M-T7 and actin (loading control).

Although MYXV is a rabbit specific poxvirus in nature, it also infects and replicates in a wide variety of cell lines derived from non-rabbit species, such as human, mouse, non-human primates, or hamster. In fact, MYXV replication is especially robust in a wide variety of human cancer cells *in vitro* [29], [30], [31], [32] or in tumor tissues within various animal models of cancer *in vivo* [33], [34], [35], [36]. We next tested the requirement of M029 for replication of MYXV in different cell types, from a variety of non-rabbit species, in culture. Both vMyx-M029KO and vMyx-M029ID viruses were unable to replicate in all human cell lines tested (eg A549, HeLa, THP1) including all cancer cells tested (not shown) and even in primary fibroblast cell lines GM02504 (Fig. 3A). In these human cell lines, both M029-minus virus constructs could bind, enter and initiate the synthesis of early viral protein (eg M-T7), however, the infection was then aborted and no synthesis of late protein (Serp-1) could be detected (Fig. 3B, top and middle panels). No defect in viral replication or protein synthesis was observed when these cell lines were infected with control vMyx-GFP (Fig. 3 A and B). This suggests that abortive infection by M029-defective MYXV in human cells was due to virus abort at some point between early and late protein synthesis. A comparable abortive infection by these two M029-defective viruses was also observed in BSC40 and NIH3T3 cell lines, which are of nonhuman primate and mouse origins, respectively (Fig. 4A, B and D). However, in baby hamster kidney BHK-21 cells, both vMyx-M029KO and vMyx-M029ID viruses were able to progress further to late stages of the viral life cycle but replicated far less efficiently compared to vMyx-GFP infection (Fig. 4C and D). This is likely linked to the delayed late viral protein synthesis from the M029-defective viruses as observed by monitoring Serp-1 protein expression (Fig. 4E top panels, compare lanes 4, 5 and 6 with 11, 12 and 13). No late protein synthesis was also observed in the M029-defective virus infected NIH3T3 or BSC40 which are permissive for the control vMyx-GFP virus (Fig. 4E and not shown).

### M029 is expressed as an early protein, is associated with virions and is localized in the nuclear and cytosolic compartments in virus-infected cells

**Figure 5:**
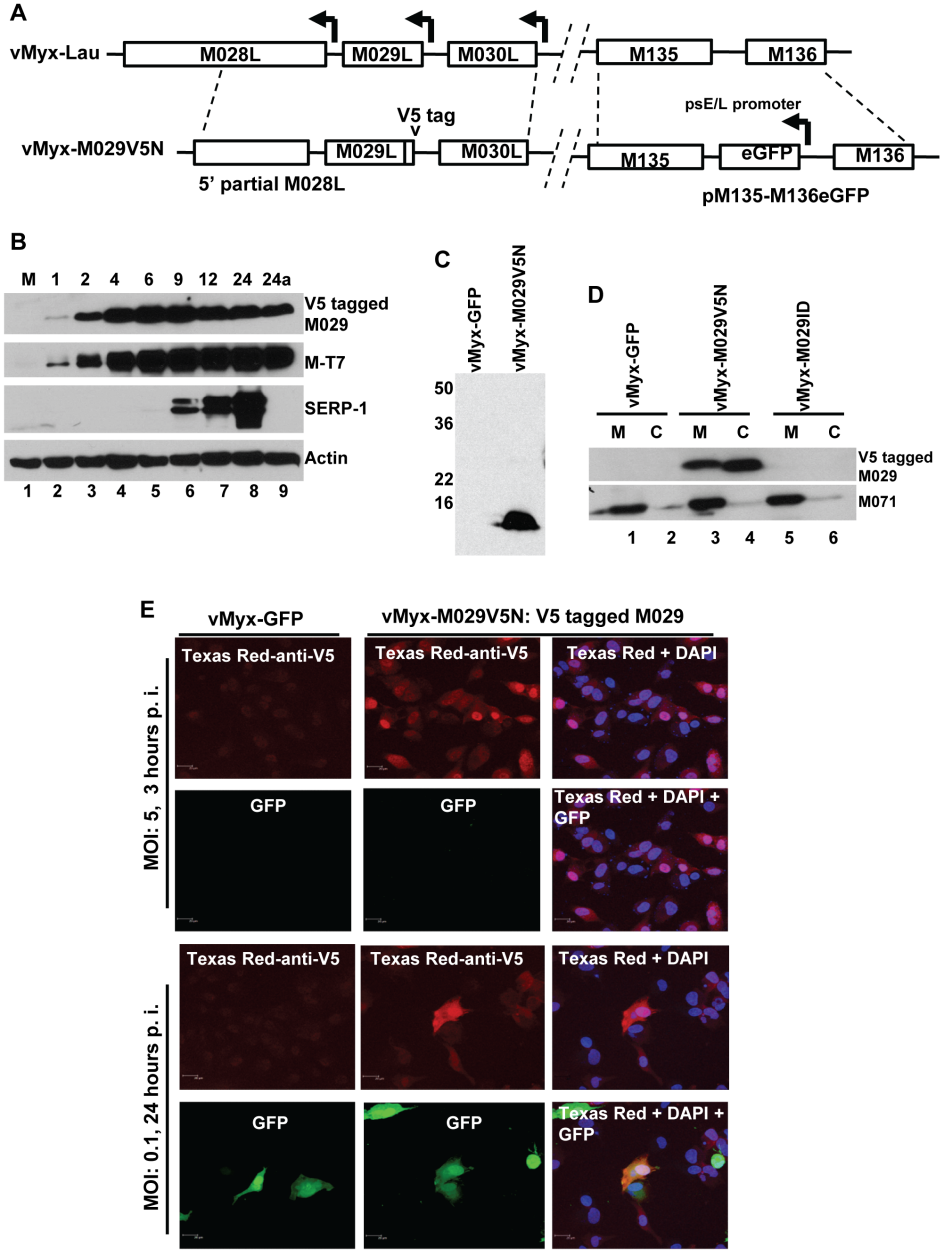
M029 is expressed as an early/late viral protein, associated with virions and is localized in the nucleus and cytoplasm of virus-infected cells. A) Construction of vMyx-M029V5N. A plasmid having N-terminal V5 sequence –tagged M029 was constructed by PCR. The final recombination plasmid has 5′ partial M028L sequence, V5-tagged M029 sequence and M030L sequence and was constructed by three-element recombination of the Multisite Gateway system. The virus also has a eGFP expression cassette inserted at the intergenic region between M135-M136 locus; B) M029 protein is expressed as an early/late gene product in infected rabbit cells. RK13 cells were left uninfected (M) (lanes 1) or were infected with vMyx-M029V5N at an MOI of 3. Cells were collected at1, 2, 4, 6, 9, 12, and 24 (lanes 2 to 8) and 24a in the presence of AraC (lane 9) h p.i. The membranes were first probed with anti-Serp-1 antibody, stripped and probed for M-T7 and actin (loading control). C) M029 is packaged into MYXV virions. Gradient purified vMyx-GFP (lane 1) and vMyx-M029V5N (lane 2) viruses were separated on 12% SDS-PAGE gels for Western blotting. V5-tagged M029 was detected using an anti-V5 antibody. D) M029 is located in the membrane and core of the MYXV virion. Gradient purified vMyxGFP (lanes 1 and 2), vMyx-M029V5N (lanes 3 and 4) and vMyx-M029ID (lanes 5 and 6) were treated with detergent and DTT to separate the membrane (M) and core components (C) of the virions. The fractions were separated on SDS-PAGE for Western blotting, followed by detection using an anti-V5 antibody, stripped and probed with anti-M071 antibody as control to detect successful separation of membrane and core fractions. E) M029 localizes to the nuclear and cytoplasmic compartments of the infected cells. RK13 cells grown on glass coverslips were infected with the indicated viruses at a MOI of 5.0 or 0.1. After 3 or 24 hr after infection, cells were fixed, permeabilized, and incubated with anti-V5 monoclonal antibody, followed by Texas Red-conjugated goat anti-mouse antibody. DNA in nuclei and viral factory was stained with DAPI (shown in blue). Cells were imaged using a Leica laser scanning confocal microscope. The localization of V5-tagged M029 is shown in red and the expression of GFP is shown in green.

In order to investigate the biological role(s) of MYXV encoded M029 protein, we created a recombinant MYXV expressing N-terminal V5-tagged M029 protein, leaving expression under the native M029 promoter (Fig. 5A). The resulting virus, vMyx-M029V5N, was used to examine M029 protein expression, cellular localization and cellular interactions in the virus-infected cells. Western blot analysis of vMyx-M029V5N infected RK13 cells demonstrated that M029 is expressed early (Fig. 5B, top panel). The treatment of infected cells with cytosine β-D-arabinofuranoside (Ara-C) (Fig. 5B, lane 9) did not affect the synthesis of M029, confirming that M029 is expressed as an early gene product. Ara-C blocked the expression of late MYXV protein Serp-1 (Fig. 5B, 3^rd^ panel), which served as a control for the outcome of inhibition of DNA replication. Based on the detection of M029 protein within the first hour of infection, we then tested whether M029 protein is associated with the input MYXV virions. We were able to readily detect V5-tagged M029 in the purified vMyx-M029V5N virions, but not in control vMyx-GFP virions (Fig. 4C). We then prepared core (C) and soluble membrane (M) fractions of purified vMyx-GFP, vMyx-M029V5N, and vMyx-M029ID viruses as previously described [37] and assessed for the presence of V5-tagged M029 protein using anti-V5 antibody. The Western blot results indicate that M029 is associated with both the core and membrane fractions of purified virions (Fig. 5D), while as a fractionation control MYXV protein M071 (an IMV surface protein) is detected only in the membrane fractions [38]. However, we were not able to detect any comparable association of E3 with VACV virions and this result is consistent with previous observations (not shown, and [39]).

We next studied the localization of M029 protein in virus-infected cells using immunofluorescence microscopy. When RK13 cells were infected with vMyx-M029V5N at an MOI of 5 for 3 hrs, V5-tagged M029 protein was detected in both the nuclear and cytoplasmic compartments of the infected cells, with some cells having relatively higher levels of M029 in the nucleus (Fig. 5E, Panels 1 and 2). We also infected RK13 cells with the same virus at an MOI of 0.1 for 24 hrs. At this time point, virus-infected cells express GFP driven by a viral early/late promoter, however there were no significant changes in the nuclear and cytoplasmic localization of V5-tagged M029 in the infected cells at the late time points (Fig. 5E, panels 3 and 4). Infection with vMyx-GFP served as a control (Fig. 5E, column 1).

### M029 interacts with PKR in a dsRNA-dependent fashion and binds an additional host protein DHX9/RHA by direct protein-protein interactions

**Figure 6:**
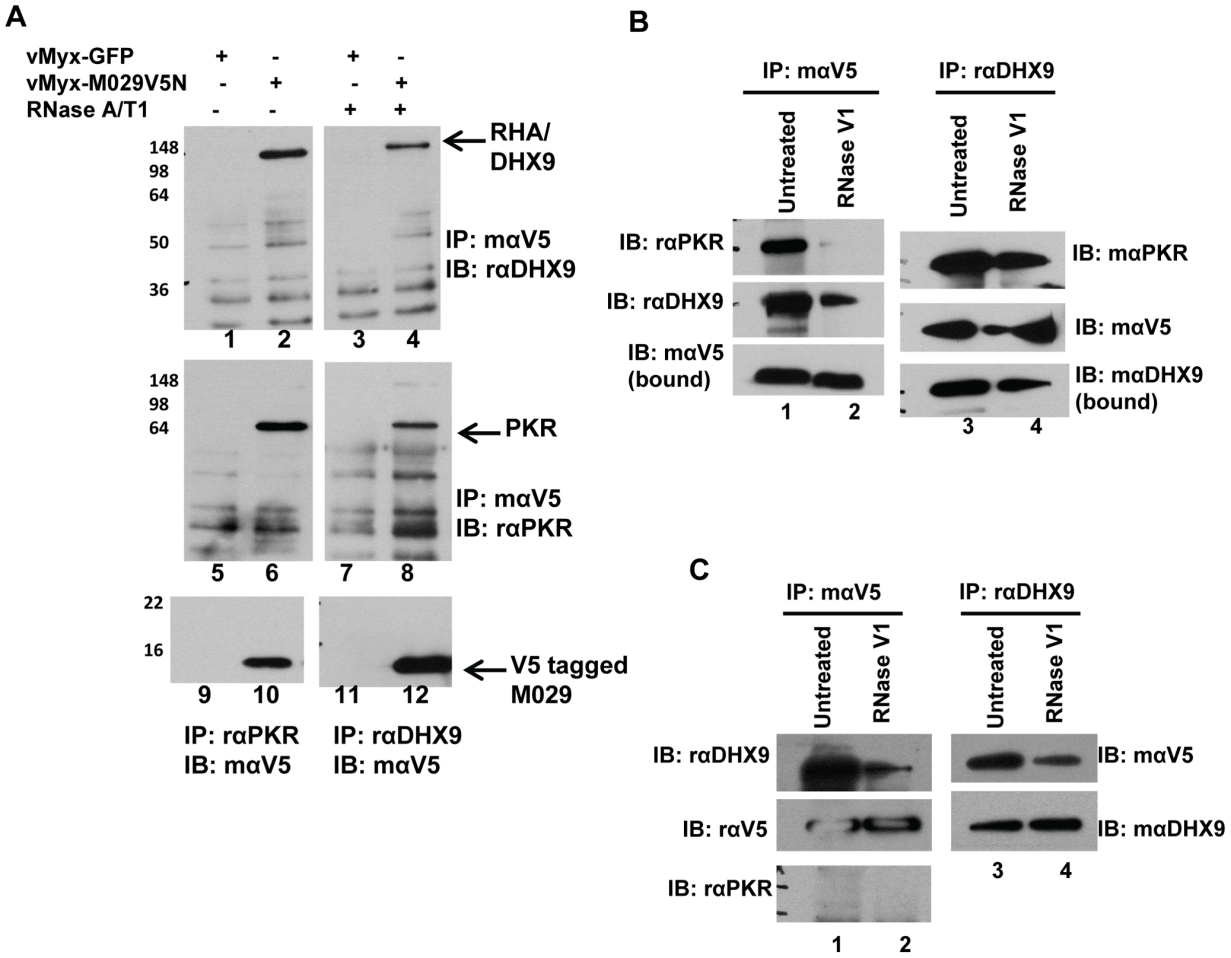
M029 interacts with human PKR and DHX9/RHA in virus-infected cells. A) HeLa cells were infected with vMyx-GFP (lanes 1, 3, 5, 7, 9 and 11) or vMyx-M029V5N (lanes 2, 4, 6, 8, 10 and 12) for 24 h. Cell lysates were untreated or treated with RNAse A/T1(50 µg/ml RNase A and 125 u/ml RNase T1) at 4°C and co-IP was performed using mouse anti-V5 antibody (lanes 1 to 8), rabbit anti-PKR (lanes 9 and 10) or rabbit anti-DHX9. Proteins associated with the complex were separated on 12% SDS-PAGE, transferred to a PVDF membrane and immunoblotted. The bots were probed with rabbit anti-DHX9 (lanes 1 to 4), rabbit anti-PKR (lanes 5 to 8) or mouse anti-V5 (lanes 9 to 12). B) M029 interaction with PKR is mediated by dsRNA but interaction with DHX9 is dsRNA independent. HeLa cell were infected with vMyxM029V5N for 24 h, cell lysates were treated with RNase V1 (10 u/ml) at 4°C over-night and co-IP was performed using mouse anti-V5 (lanes 1 and 2) or rabbit anti-DHX9 (lanes 3 and 4). After transfer the blots were probed with different antibodies. C) M029 interaction with DHX9 is independent of PKR. HeLa cell lines with constitutive knock down of PKR (HeLa shPKR, described in figure 9) were infected with vMyx-M029V5N for 24 h, cell lysates were treated with RNase V1 at 4°C overnight and co-IP was performed using mouse anti-V5 (lanes 1 and 2) or rabbit anti-DHX9 (lanes 3 and 4). After transfer the blots were probed with different antibodies.

We next investigated whether M029 protein interacted with any specific host protein(s) in virus-infected cells that might regulate MYXV replication in diverse cell types, given that in the absence of functional M029 expression, MYXV became nonpermissive in so many diverse mammalian cell types. For this proteomic study, we primarily focused on virus-infected human cells in order to identify any potential M029 interacting cellular proteins that potentially regulate MYXV tropism. Human A549 cells were infected with vMyx-GFP or vMyx-M029V5N viruses, the protein complexes bearing V5-tagged protein were isolated using anti-V5 antibody by co-IP and unique protein bands present in vMyx-M029V5N samples were identified by mass spectrometry. In order to identify all the potential M029 interacting proteins, proteomic analysis was performed with samples that were not treated with RNase V1, a ribonuclease that cleaves double-stranded RNAs. Multiple distinct co-precipitating proteins were identified from the vMyx-M029V5N infected sample (Table 1). However, when the same samples were treated with RNase V1, many of the protein bands were disappeared (data not shown). One of the identified proteins was RNA Helicase A (RHA), also known as DHX9, which was consistently present in the immunoprecipitates with V5-tagged M029. This interaction of M029 with DHX9 was confirmed from multiple human virus-infected cells, including HeLa, A549 and THP1 cell lines (Fig. 6A lane 2 and data not shown). The presence of PKR was also detected in the same anti-V5 co-IP samples by Western blot using anti-PKR antibody (Fig. 6A, lane 6). The interactions between V5-tagged M029 and DHX9 or PKR were also confirmed by co-IP using anti-DHX9 or anti-PKR antibody, respectively and detection of tagged M029 using anti-V5 antibody (Fig. 6A lanes 10 and 12). Next we tested whether interaction of M029 with PKR or DHX9 might be dependent on linkage via dsRNA, since both the host proteins and M029 all possess dsRNA-binding domains. Treatment with ssRNase, RNaseA/T1, did not affect the interactions of M029 with either DHX9 or PKR (Fig. 6A lanes 4 and 8). In contrast, when the lysates were pre-treated with RNase V1, which cleaves dsRNA substrates, the amount of PKR that interacted with M029 using dsRNA was no longer observed. However, DHX9 was still detected in the samples precipitated with anti-V5 after being treated with RNase V1 (Fig. 6B compare lanes 2 and 4). These results suggest that M029 protein interacts with PKR in a dsRNA-dependant manner but interacted with DHX9 independently of dsRNA. We also confirmed protein-protein interactions between M029 and DHX9 in the absence of PKR, using the cell lysates prepared from PKR stable knockdown HeLa cells (Fig. 6C).

**Table 1:** Cellular proteins associated with V5-tagged M029 immunoprecipitations.

Protein name	Mass (Da)
ATP-dependent RNA helicase/DHX9	142,140
Long isoform of splicing factor, proline and glutamine rich	76,149
dsRNA-binding protein staufen homolog 2	59,000
Heterogeneous nuclear ribonucleoproteins A/B	35,967
KH domain containing RNA binding signal transduction-associated protein 1	48,228
Isoform A1-B of heterogeneous nuclear ribonucleoproteins A1	38,747
Heterogeneous nuclear ribonucleoproteins A2/B1	37,430
ADP/ATP translocase 2	32,853
60S ribosomal protein L7	29,227
Single stranded DNA binding protein	17,259
60S ribosomal protein L30	12,784

### PKR is activated in cells infected with M029-minus MYXV leading to an abortive infection

**Figure 7:**
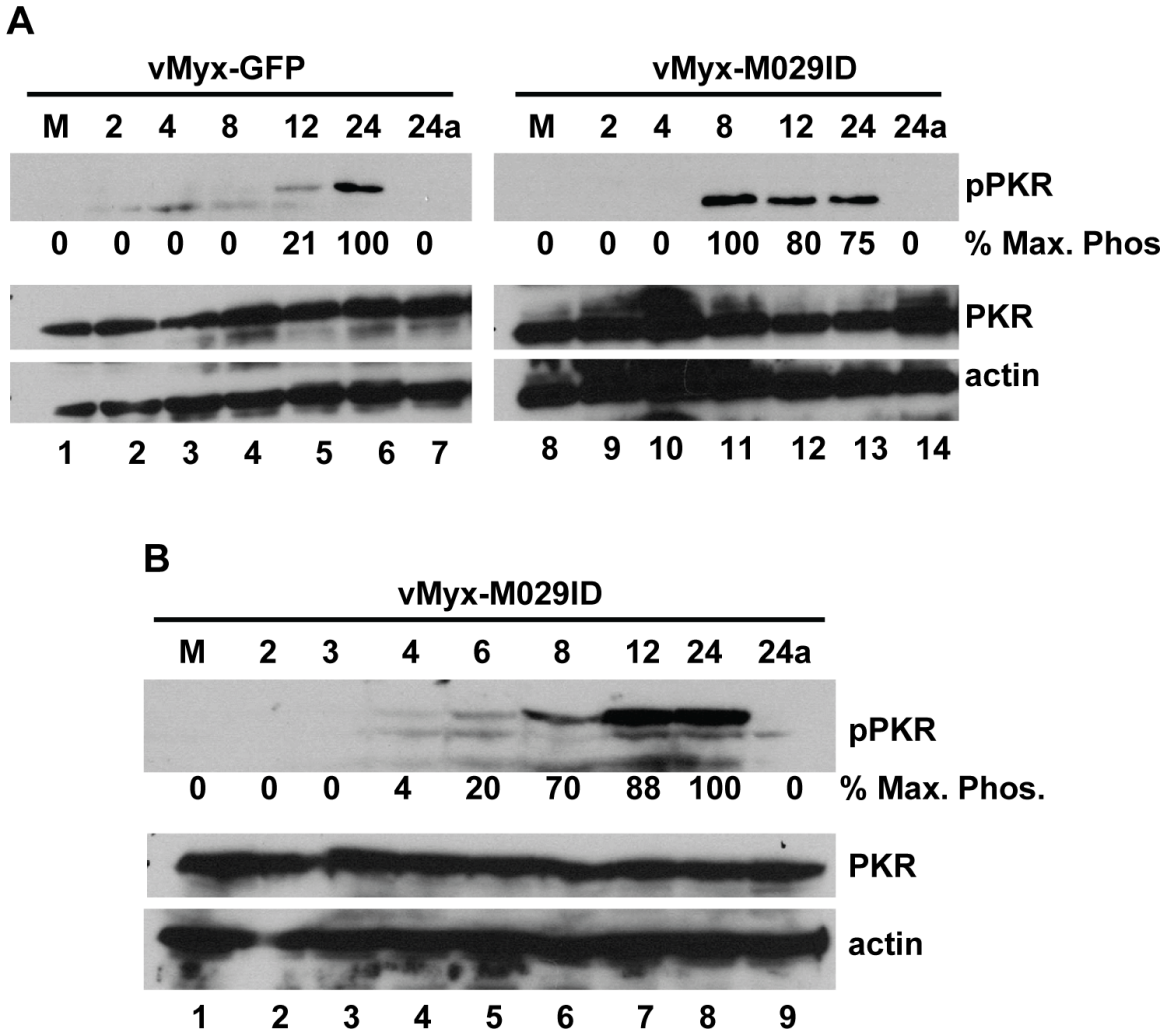
M029 blocks early activation of PKR signaling in MYXV-infected cells. A) HeLa cells were left uninfected (M) (lanes 1 and 8) or infected with vMyx-GFP (lanes 2 to 7) or vMyx-M029ID (lanes 9 to 14) at an MOI of 3. Cells were collected at 2 (lanes 2 and 9), 4 (lanes 3 and 10), 8 (lanes 4 and 11), 12 (lanes 5 and 12), 24 (lanes 6 and 13) and 24a in the presence of AraC (lanes 7 and 14) h p.i. The membranes were first probed with anti phospho PKR (pPKR) antibody, stripped and probed for PKR and actin (loading control). B) Western blots showing expression of pPKR, PKR and actin in vMyx-M029ID infected samples collected after 2, 3, 4, 6, 8, 12, 24 hrs and 24a in the presence of AraC from HeLa cells. The relative level of phosphorylated PKR is indicated.

We have examined the role of PKR activation in the regulation of MYXV infection in the absence of functional M029 protein. Infection of human cells with vMyx-M029ID or vMyx-M029KO viruses activated PKR signaling soon after infection, as compared to control wildtype-MYXV infection. In the M029-minus virus-infected cells, high level of phospho-PKR was detected 8 hpi (Fig. 7A, compare lanes 4 to 6 with lanes 11 to 13) and could be observed even at 4 hpi (Fig. 7B, lanes 4 to 8). No phospho-PKR was detected before 12 hpi in the samples infected with control vMyx-GFP, suggesting that repression of PKR activation by M029 is key for permissive MYXV replication. Importantly, we noticed that treatment of virus-infected cells with AraC prevented the phosphorylation of PKR induced by both M029-minus viruses. This suggests that the dsRNA ligands, which are preferentially synthesized in poxvirus-infected cells following DNA replication, are necessary for PKR phosphorylation and are sequestered by M029 (Fig. 7A and B).

### PKR knockdown in human cells rescues M029-minus virus replication in human cells

**Figure 8:**
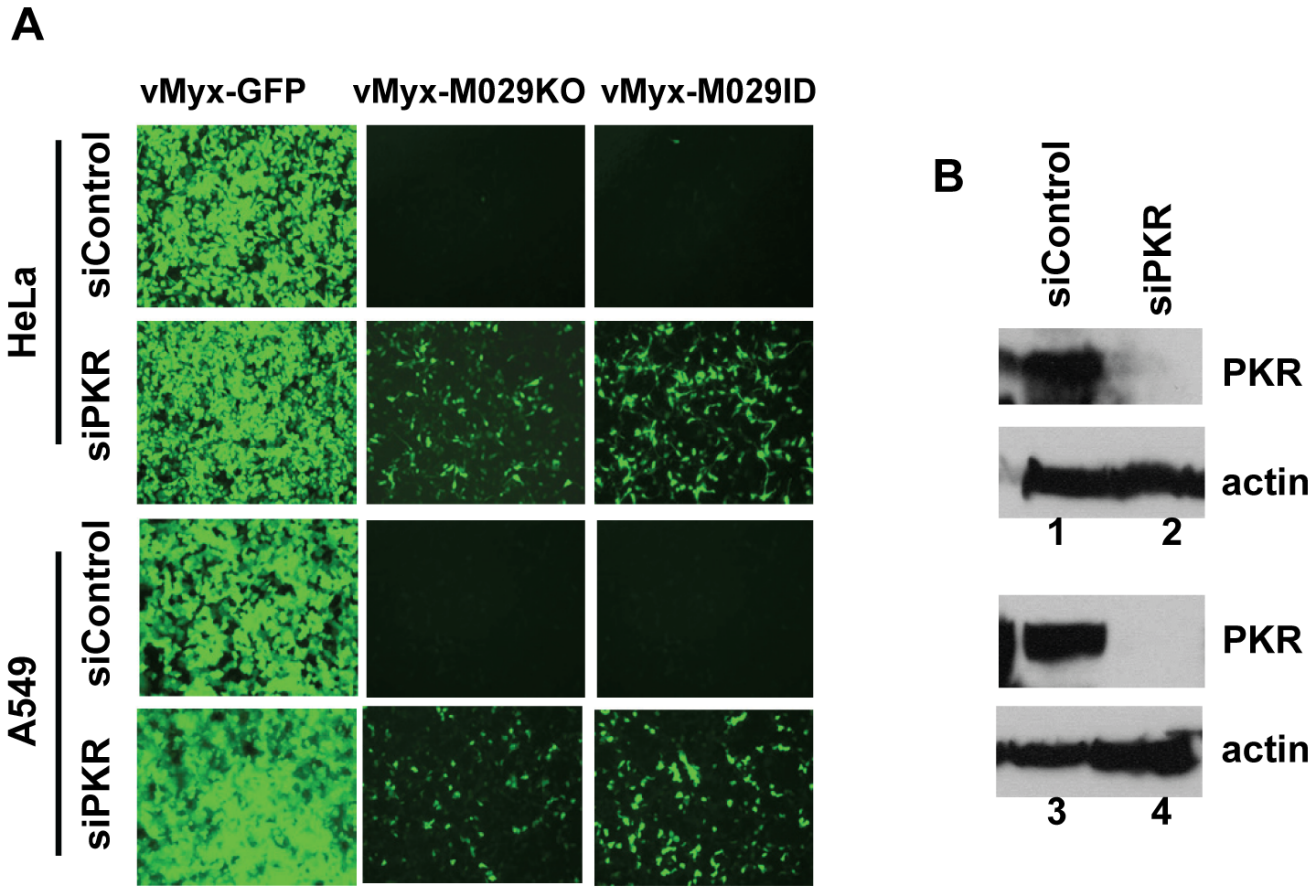
Transient knocking down of PKR by siRNA can rescue the replication of vMyx-M029KO and vMyx-M029ID. A) HeLa and A549 cells were transiently transfected with control siRNA (panels in rows 1 and 3) or siRNAs targeting human PKR (final concentration of 100 nM) (panels in rows 2 and 4) for 48 h before infection with vMyx-GFP (column 1), vMyx-M029KO (column 2) and vMyx-M029ID (column 3) at an MOI of 3 for 48 h. B) Western blot of siRNA transfected cell lysates showing the level of PKR knock down in HeLa (Lane 2, panel 1) and A549 cells (lane 4, panel 3).

**Figure 9:**
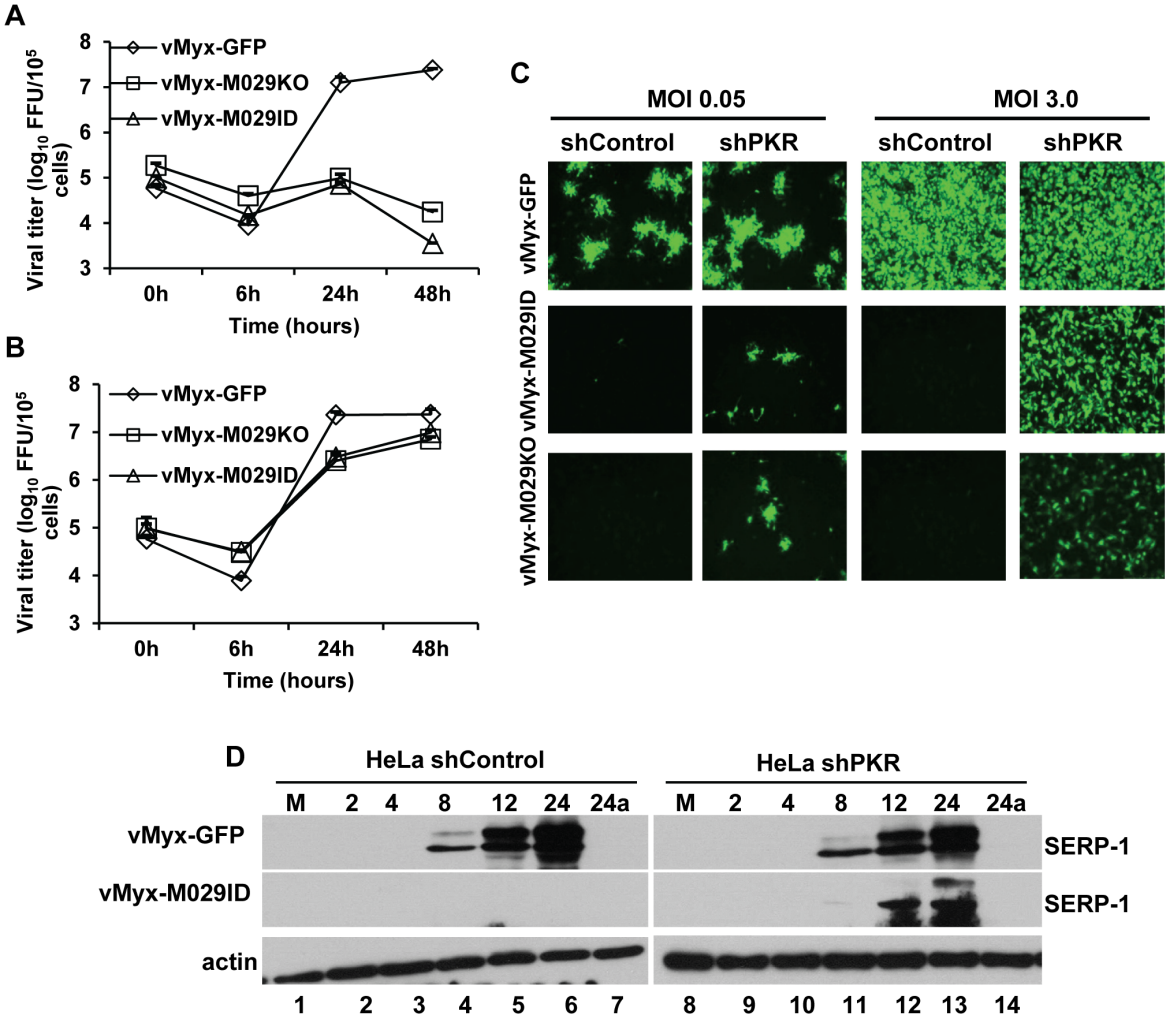
Stable knock down of PKR can rescue the replication of M029-defective MYXV in human cells. HeLa cell lines with constitutive knockdown of PKR (shPKR) were constructed by infecting parental HeLa with a lentivirus packaged with shRNAs targeting PKR mRNA. A control HeLa cell line (shControl) was also constructed by infecting HeLa cells with a lentivirus packaged with control shRNAs. Single step growth curves of MYXV infection in A) shControl B) shPKR cells. The indicated cells were infected with vMyx-GFP, vMyx-M029KO or vMyx-M029ID at an MOI of 5, and then cells were collected at 0, 6, 24, and 48 h p.i. The virus titers were determined in triplicate following serial dilution onto RK13-E3 cells. Data are representative of three independent experiments. C) Fluorescence microscope images of infected shControl (columns 1 and 3), and shPKR (columns 2 and 4) cells. The images were taken using an inverted fluorescence microscope using a lens with ×10 magnification at 48 h p.i. The cells were infected with a low MOI of 0.5 (columns 1 and 2) and high MOI of 3.0 (columns 3 and 4) with vMyx-GFP (top panels), vMyx-M029ID (middle panels) and vMyx-M029KO (bottom panels). D) Expression of viral early (M-T7) and late (Serp-1) proteins in the infected shControl and shPKR cell lines. Cells were left uninfected (M) (lanes 1 and 8) or were infected with vMyx-GFP (top panels) or vMyx-M029ID (middle panels) at an MOI of 3. Cells were collected at 2, 4, 8, 12, 24 and 24a in the presence of AraC (lanes 7 and 14) h p.i. The membranes were first probed with anti-Serp-1 antibody, stripped and probed for actin (loading control).

Identification of PKR as an M029 interacting host protein, and the activation of PKR in virus-infected cells in the absence of M029 expression, both suggest that PKR can play significant role(s) in the innate anti-viral response initiated in PKR-competent mammalian cells in response to MYXV infection. To study the direct role of PKR in MYXV infection, we first knocked down PKR expression in various human cells by transient transfection of an siRNA pool targeting human PKR. Knocking down PKR expression caused significant increase in the replication of both vMyx-M029KO and vMyx-M029ID viruses in all tested human cell lines such as A549, HeLa, THP1, and primary human fibroblast cells GM02504 (Fig. 8A, and not shown). In order to further analyze the role of PKR and achieve more consistent gene expression knockdown, a lentivirus containing shRNAs targeting PKR was used to construct human cell lines with constitutively reduced PKR protein levels. In PKR-knockdown HeLa cells (HeLa shPKR), the replication of wildtype MYXV, vMyx-GFP was unchanged (Fig. 9 A and B). In these PKR knockdown HeLa cells, the replication of both vMyx-M029KO and vMyx-M029ID viruses increased about two logs, as monitored by single step growth curves (Fig. 9B). The replication of M029-defective viruses in HeLa shPKR cells also correlated with the formation of viral foci when infected with low MOI (Figure 9C, column 2) and also restored synthesis of late viral protein in the infected cells (Fig. 9D compare lanes 12 and 13 with lanes 5 and 6).

### MYXV sensitivity to type I IFN is enhanced in the absence of functional M029

**Figure 10:**
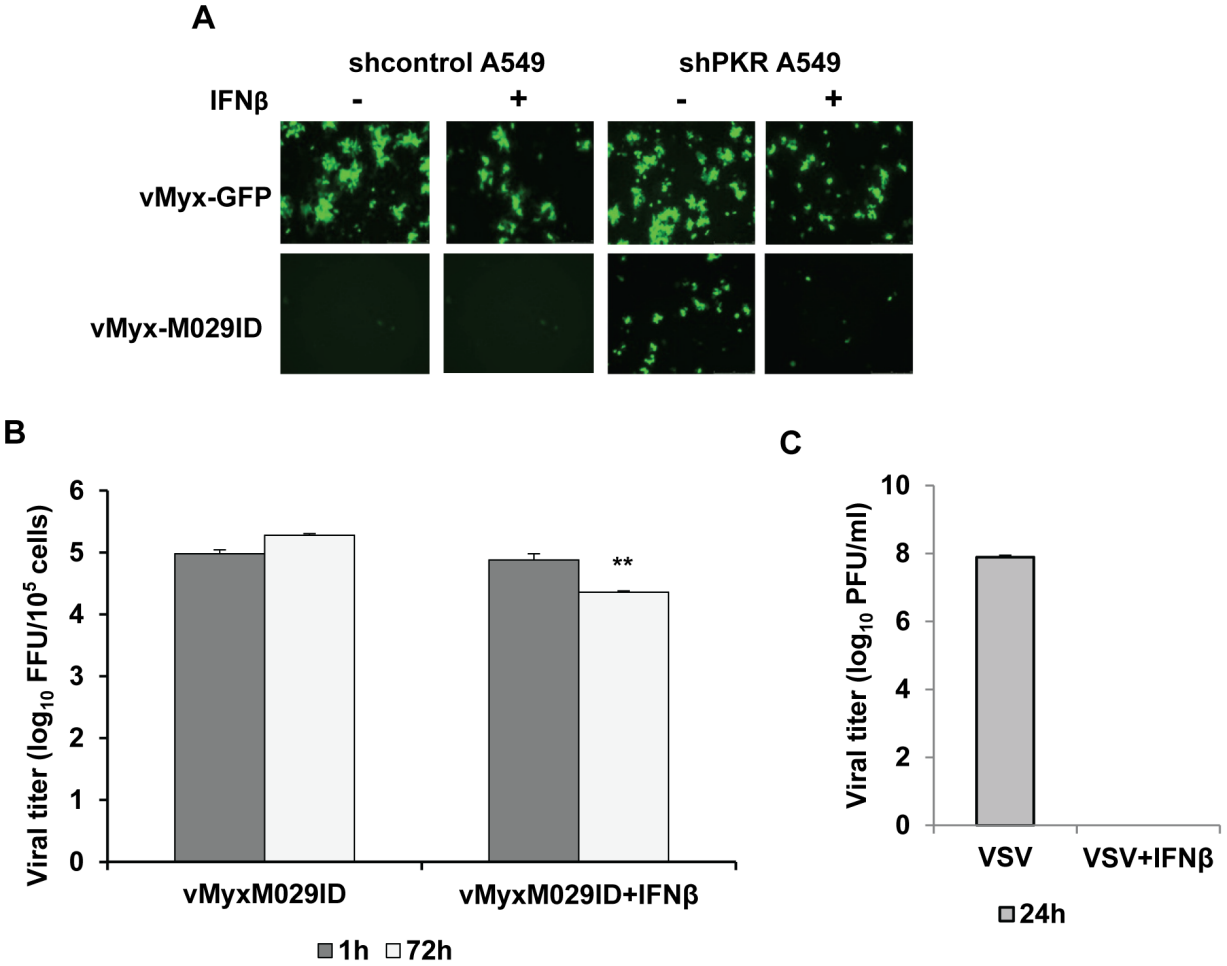
Absence of M029 enhances MYXV sensitivity to Type I IFN. Human A549 cell lines with constitutive knockdown of PKR (shPKRA549) or a control (shControl A549) were constructed as described for HeLa. The cells were infected with vMyx-GFP (top panel) or vMyx-M029ID with or without treatment of human IFNβ (1000 U/ml). A) Fluorescence images were taken after 48 h p.i. with an MOI of 0.1. B) Virus was tittered after 1 and 72 h of p. i. using RK13-E3 cells. C) A549 cells were infected with VSV with an MOI of 0.1 with or without treatment of human IFNβ (1000 U/ml). Virus was tittered after 24 h p.i. using A549 cells to determine the titer. Data are mean ± SD of triplicate samples from one experiment and are representative of two independent experiments. **P<0.01 compared with vMyx-M029ID infection.

The members of E3 family of proteins are believed to be key players in mediating resistance against IFN [25]. Using M029-minus mutants of MYXV, we have tested whether M029 plays a functional role in resistant to type I IFN in human A549 cells, which respond robustly to type I IFN [40]. We constructed stable PKR knockdown A549 cells using lentiviruses containing shRNAs targeting PKR. In the A549 or ShControl A549 cells, treatment with IFNβ resulted in the formation of smaller foci by vMyx-GFP and slowed the replication kinetics of M029-expressing MYXV (Fig. 10A and not shown). However, significant differences (*P*<0.01) were observed in the PKR knockdown A549 cells. In these shPKR-A549 cells, the rescued replication of M029-defective virus, vMyx-M029ID, was almost abolished in the presence of IFNβ (Fig. 10A bottom panels). When progeny virus was titered, we also observed significant reduction in the vMyx-M029ID virus production in the PKR-knockdown cells that had been interferon-treated (Fig. 10B, 72 hpi).

### DHX9/RHA is required for replication of MYXV in human monocytic cells

**Figure 11:**
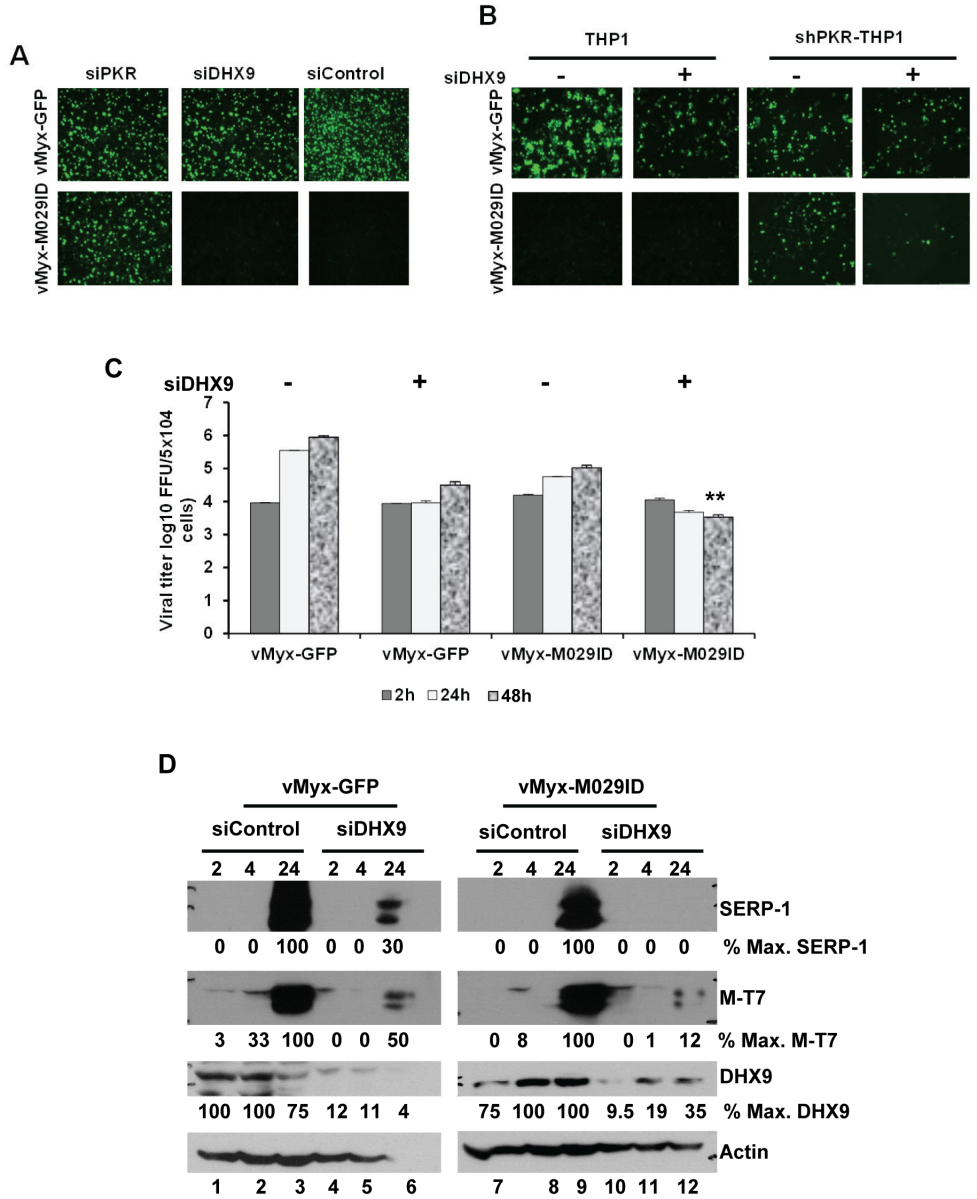
RHA/DHX9 is required for the rescue of M029-defective MYXV replication in human cells. A) Transient knockdown of endogenous RHA/DHX9 alone did not induce rescue of M029 mutants in THP1 cells. THP1 cells were transiently transfected with PKR siRNA (left column), DHX9 siRNA (middle column) or control siRNA (right column) for 48 h before infection with vMyx-GFP (top panels) or vMyx-M029ID (bottom panels) at an MOI of 3 for 48 h. The images were taken using an inverted fluorescence microscope. B) Transient knock down of DHX9 in shPKR THP1 cell lines prevent the rescue of M029 defective viruses. THP1 cell lines with constitutive knockdown of PKR (shPKR-THP1) were constructed by infecting parental THP1 with a lentivirus packaged with shRNAs targeting PKR mRNA. ShControl and ShPKR-THP1 cells were transiently transfected with DHX9 siRNA or control siRNA for 48 h before infection with vMyx-GFP or vMyx-M029ID at an MOI of 3. Fluorescent microscope images of vMyx-GFP (top panels) or vMyx-M029ID (bottom panels) after 48 h p.i. C) vMyx-GFP or vMyx-M029ID infected cells were collected at 2, 24 and 48 h p.i. for titration of virus production using RK13-E3 cells. Data are mean ± SD of triplicate samples from one experiment and are representative of two independent experiments. ** *P*<0.01 compared with un-transfected samples. D) vMyx-GFP (lanes 1 to 6) or vMyx-M029ID (lanes 7 to 12) infected shPKR THP1 cells after DHX9 or control siRNA transfection were harvested at 2, 4 and 24 h p.i and proteins were detected for Serp-1, M-T7, DHX9 and actin (as loading control). The relative level of SERP-1, M-T7 and DHX9 protein expression is indicated.

In order to investigate the role of RHA/DHX9 in MYXV replication in human cells, we used siRNA to knockdown the expression of DHX9 in various human cells. Knocking down RHA/DHX9 protein expression in HeLa, A549 or THP1 cells did not rescue the M029 defective virus infection, nor were any significant changes in the replication of wildtype-MYXV detected (Fig. 11A and not shown). Since DHX9/RHA has also been reported to interact with PKR ([41] and our results), we then decided to investigate whether it has any role associated with PKR functions. We therefore transiently knocked down the expression of RHA/DHX9 in the PKR stable knockdown shPKR human cell lines. In the shPKR-THP1 cell lines, where M029 defective virus replication has been rescued, we observed significant decrease (*P*<0.05) in the replication of vMyx-M029ID compare to vMyx-GFP, when RHA/DHX9 was knocked down (Fig. 11B). This was reflected by a decrease in the progeny virus titer when monitored during a one-step virus replication cycle (Fig. 11C). Knocking down RHA/DHX9 in the stable shPKR-THP1 cells also reduced the synthesis of both early (M-T7) and late (Serp-1) viral proteins in the vMyx-M029ID infected cells (Fig. 11D). However, the synthesis of both viral early and late mRNAs remained unaffected in these cells, suggesting that RHA/DHX9 may have role in viral protein synthesis (Figure S4). Thus, in stark contrast to PKR, whose activation was inhibitory to MYXV in all the human cells tested, RHA/DHX9 is required for optimal MYXV replication in human THP-1 myeloid cells when PKR was depleted or absent.

### M029 is essential for pathogenesis of MYXV in susceptible European rabbits

**Figure 12:**
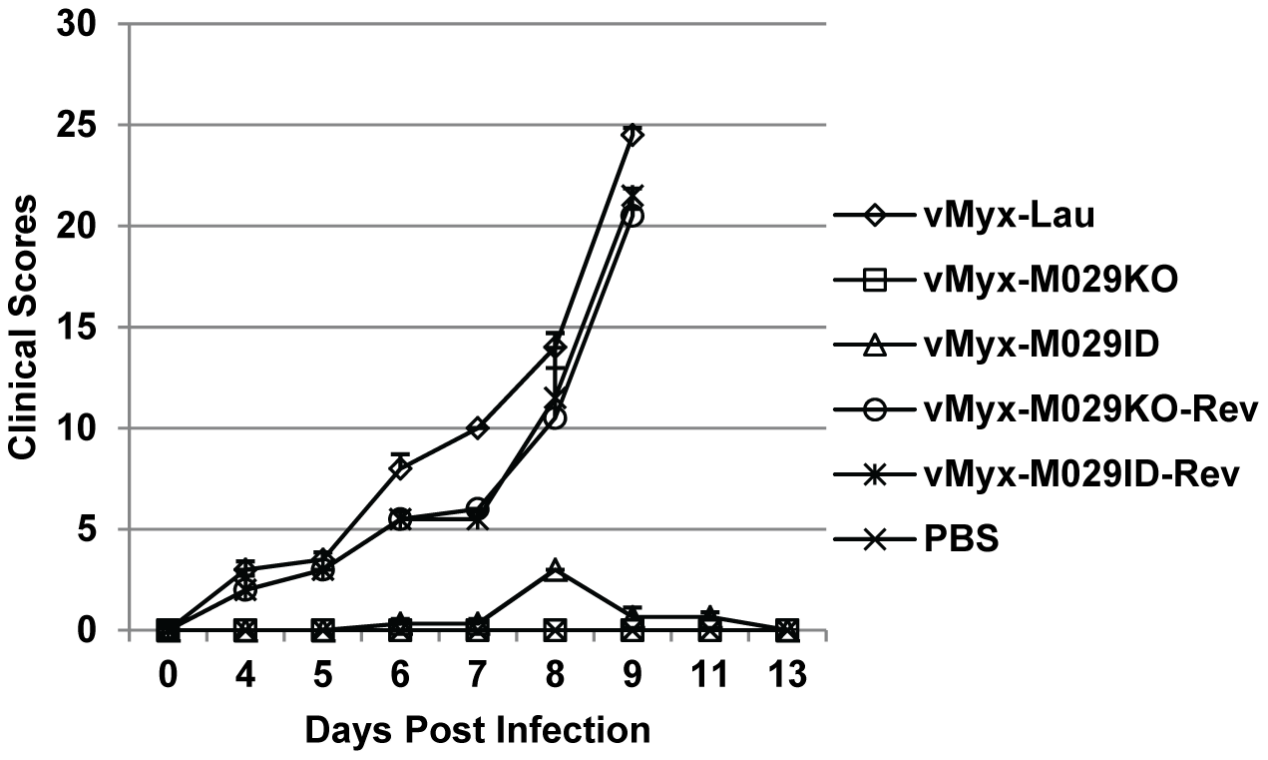
M029 is essential for MYXV pathogenesis in European rabbits. Both vMyx-M029KO and vMyx-M029ID viruses can no longer cause myxomatosis in European rabbits. The overall physical condition and disease progression were evaluated daily using a clinical score system, and the average daily score for the rabbits representing a particular treatment group was calculated and plotted.

We next examined the role of M029 in the pathogenesis of MYXV in the European rabbits, where MYXV causes lethal disease myxomatosis. Viruses (1000 FFU) were delivered by intradermal route of inoculation to the flanks of susceptible European rabbits. In this study, we used mock infected (PBS), wild-type parental MYXV Lausanne strain (vMyx-Lau), or revertant viruses containing a rescued intact M029 gene as controls. Infection with M029-revertant viruses showed similar progression of myxomatosis and same mortality rates (100%) as that caused by the parental wild-type virus (Table 2 and Fig. 12). The vMyx-M029KO and vMyx-M029ID viruses induced either no, or very mild, primary lesions at the primary inoculation site, and no observable disease indications were detected at all throughout the entire course of observation. All the animals infected with these M029-mutant viruses survived the initial infection with no noticeable symptoms (Table 2). These observations suggest an essential role for M029 in the infections and pathogenesis of MYXV in the susceptible European rabbit hosts. We also tested whether abortive infection of M029 mutant viruses might function as potential vaccines for myxomatosis, as caused by the wild-type vMyx-Lau infection. However, we observed low but variable protection results, suggesting that the M029-mutant infections aborted so quickly at the primary site of inoculation that very little in the way of acquired immune responses were generated.

**Table 2:** Pathogenesis of vMyx-M029KO and vMyx-M029ID in New Zealand White (NZW) rabbits.

Day	vMyx-Lau/vMyx-M029KO-Rev/vMyx-M029ID-Rev	vMyx-M029KO	vMyx-M029ID
0	For each of the indicated viruses two female NZW rabbits were inoculated intradermally with 10^3^ FFU/0.1 ml.	Four female NZW rabbits were inoculated intradermally with 10^3^ FFU/0.1 ml.	Three female NZW rabbits were inoculated intradermally with 10^3^ FFU/0.1 ml.
4	All the rabbits had red and swollen primary lesion with an average diameter of 2.5 cm	No sign of primary lesion in all four rabbits	One rabbit had redness at the inoculation site
5	All the rabbits developed secondary lesions on eyes and ears; satellite lesions detected surrounding the primary lesion	No sign of primary lesion in all four rabbits	Primary lesion became red and mild swollen in one rabbit
6	Primary lesion red, swollen, and necrotic; numerous secondary lesions were detected on eyes, ears and nose in all the rabbits	No sign of primary lesion in all four rabbits	Another rabbit had redness at inoculation site
7	Primary lesion increased in diameter to 3.5 cm; eyes, ears, nose, mouth are swollen in all the inoculated rabbits	No sign of primary lesion in all four rabbits	All three rabbits had red and mild swollen primary lesion
8–9	All the rabbits had primary lesions which were 4.5 to 5.5 cm; severe signs of myxomatosis; rabbits were sacrificed due to severity of the symptoms	Two rabbits had red and mild swollen area where virus inoculated	Primary lesion red and swollen, diameter 1 cm; few secondary lesions detected on ears and nose in all three rabbits
11–12		Only one rabbit had small red, swollen area	All three rabbits had primary lesions which were red, swollen, necrotic; no secondary lesions
19–20		Rabbits were re-challenged with vMyx-Lau with 10^3^ FFU/0.1 ml. Three out of four rabbits were protected from myxomatosis after re-challenge.	Rabbits were re-challenged with vMyxLau with 10^3^ FFU/0.1 ml. No sign of myxomatosis detected in the re-challenged rabbits.

## Discussion

We have demonstrated that M029 is both a critical host range factor for MYXV replication in a wide spectrum of diverse mammalian cell types as well as a critical virulence factor for myxomatosis in the European rabbits. Our inability to isolate pure recombinant M029-defective MYXV constructs using any of the standard mammalian cell lines where the parental MYXV can robustly replicate indicated that M029 plays essential function(s) in MYXV replication for most mammalian cells. The M029-minus viruses were only successfully isolated away from complementing parental MYXV and propagated as pure clones in cell lines that stably express VACV E3, a complementing comparable poxvirus protein. The purified vMyx-M029KO and vMyx-M029ID viruses that were grown in these E3-complementing RK13 cells exhibited severe defects in replication in essentially all established mammalian cell lines originated from diverse species, where the parental wildtype-MYXV replicates permissively. The most profound tropism defect of the M029-mutant viruses was their complete inability to replicate in any of the tested cells derived from humans (eg HeLa, A549, THP-1, various cancer cell lines, primary fibroblasts, etc), non-human primates (eg BSC40) and mouse (eg NIH3T3) (Fig. 3 and 4). In these cell lines, no expression of viral late proteins was detected in the absence of M029 expression. On the other hand, abundant expression of early viral proteins was detected from M029-minus viruses at a comparable level with wildtype-MYXV infection (Fig. 3B), suggesting that there was no defect in virus binding, entry, or early viral gene expression. Both vMyx-M029KO and vMyx-M029ID viruses were also unable to replicate in rabbit RL5 T lymphocytes, and although they were capable of at least some detectable progeny virus synthesis in RK13 cells, replication kinetics were reduced and cell-cell transmission was compromised. Slower replication kinetics of M029-defective viruses was also observed in hamster BHK21 cell lines. In both RK13 and BHK21 cells, the synthesis of viral late protein was significantly delayed, which resulted in a reduced progeny virus production at 12 hpi when tested by single step growth curve analysis. It is possible that the level of dsRNA produced by the viruses in these semi-permissive cell lines is lower than the completely nonpermissive cell lines, as has been suggested for VACV [16].

The deletion or insertional disruption of M029 also resulted in the elimination of essentially all disease symptoms associated with myxomatosis in susceptible rabbits. This extreme attenuation was associated with the absence of obvious viral spread and extreme reductions in the replication of virus at the primary site of infection. Following full recovery of the M029-mutant infected animals, very variable levels of protection was generated against subsequent challenge by wildtype MYXV, suggesting that virus replication at the primary inoculation site was likely aborted quickly before the elements of acquired immune responses were significantly engaged.

Analyzing the distribution of V5-tagged MYXV M029 protein revealed that M029 was expressed at an early time points of infection and was packaged into infectious virions (Fig. 5), which suggests a critical role of M029 in the modulation of host innate sensing/signaling pathways during early stages of infection. This observation is particularly important because VACV E3 has not been reported in virions and we were also unable to detect E3 in purified VACV particles using comparable strategy used for vMyx-M029V5N ([39], and data not shown). Localization studies using immunofluorescence microscopy indicated that M029 protein is localized to both the nuclear and cytosolic compartments of MYXV-infected cells. This same cellular localization of M029 was also observed when V5-tagged M029 was transiently expressed in uninfected cells using plasmids under CMV promoter (data not shown), suggesting that nuclear-cytoplasmic dual localization is not dependent on viral infection. Like M029, VACV E3 protein is also localized in both the nucleus and cytoplasm, however, the nuclear localization of E3 is dependent on the N-terminal 83 amino acid residues, which are missing in the M029 protein [26], [42]. This suggests that M029 may have acquired a different mechanism for nuclear localization.

The major role of VACV E3 is thought to antagonize the PKR-mediated anti-viral functions of the infected host cell, which are upregulated by IFN and activated by dsRNA [4]. eIF2α is a primary cellular substrate that becomes phosphorylated in cells infected with E3-minus VACV, which then leads to the global inhibition of host and viral protein synthesis, the induction of apoptosis and eventual inhibition of virus replication. Infection with VACVΔE3 resulted in the phosphorylation of both PKR and eIF2a, however the defect of VACVΔE3 can be rescued in PKR-deficient HeLa cells [43]. In the PKR-deficient cells, late viral protein synthesis was restored and virus-induced apoptosis was also abolished, which resulted in productive virus replication even in the absence of E3 expression. PKR is one of the host innate immune proteins that undergo rapid evolution in order to escape being targeted by viruses capable of anti-PKR strategies, such as poxviruses [44].

Based on the profound host range phenotype of M029-defective viruses in a wide spectrum of mammalian cells, we created MYXV that expressed V5-tagged M029 and performed co-IP and mass-spectrometry to identify putative host binding partner proteins that might be functionally targeted by M029. Based on the previous report that PKR interacts with VACV E3, we first identified cellular PKR as an M029 interacting protein in various human cells. The interaction of PKR and M029 was validated by co-IP assay in virus-infected mammalian cells with either V5 or PKR antibody (Figure 6A). Significantly, this PKR-M029 protein interaction was eliminated by digestion with RNaseV1, which cleaves dsRNA, but not with RNaseA/T1 that targets only ssRNA, suggesting that this interaction requires dsRNA. Since both PKR and M029 possess dsRNA-binding domains, it is presumed that these proteins become linked via dsRNA bridging. VACV E3, on the other hand, has been reported to have direct protein-protein interaction with PKR; however, the samples were not treated with an RNase that specifically cleaves dsRNA [45], [46]. Although M029 interacts with PKR indirectly via dsRNA, this interaction is still biologically relevant, since the replication of M029-minus MYXV viruses were specifically rescued in PKR knockdown cells. In stable PKR knockdown human cells, the synthesis of viral late proteins was fully restored and allowed complete replication and progeny virus formation by vMyx-M029KO and vMyx-M029ID (Fig. 9), which demonstrates that PKR is a major functional target for M029 host range functions, as it is for VACV E3.

In addition to PKR, we also identified several other cellular RNA binding proteins that were consistently present in the co-IP samples for V5-tagged M029 in virus-infected human cells. Among these, we were particularly interested in investigating the potential interaction of M029 with RHA, a cellular RNA helicase, also known as DHX9. RHA/DHX9 is a 130 kDa protein that belongs to the DEXD/H box family of proteins, which can unwind both double-stranded RNA and DNA, and can also regulate cellular processes such as pre-mRNA splicing, ribosome biogenesis, transcription, RNA nuclear export, and translation initiation [47], [48], [49]. RHA/DHX9 is also associated with regulating the replication efficiencies of various RNA viruses, including HIV-1, HCV, FMDV and influenza A [50], [51], [52], [53]. These viral regulatory functions of RHA/DHX9 are mediated by interactions with unique viral proteins, for example, the Gag protein of HIV and NS1 protein of Influenza A virus [53], [54]. DHX9 also function as a dsRNA sensor in selected cell types [55]. In one recent report, it was demonstrated that NS1 can rescue VACVΔE3 virus replication, at least *in vitro* [24], and thus we postulated that RHA/DHX9 might also have potential role(s) in some aspect of poxvirus tropism. VACV E3 has also been reported to interact with a wide spectrum of host proteins, including RHA/DHX9, but the functional significance of this particular protein interaction has not been reported [56]. The interaction of DHX9/RHA with V5-tagged M029 protein was validated in virus-infected cells by co-IP assay using either anti-RHA/DHX9 or V5 antibody. Interestingly, unlike the PKR-M029 interaction, the RHA-M029 protein interaction was not eliminated by digestion of dsRNA with RNaseV1 (Fig. 6A and B), suggesting that interaction between RHA/DHX9 and M029 is through direct protein-protein interactions. RHA/DHX9 has also been reported to interact with PKR, and is in fact a substrate of activated PKR kinase [41]. In the virus infected cells we have not observed any alteration in the nuclear localization of DHX9 (data not shown). We therefore assessed the potential biological significance of RHA/DHX9 interaction with M029, and discovered that this interaction regulated virus host range in a very cell-specific fashion. Knockdown of RHA/DHX9 gene expression in human THP-1 myeloid cells that had been depleted of PKR reduced the replication and viral protein synthesis for both the M029-expressing vMyx-GFP and significantly for the M029-defective viruses, indicating that RHA/DHX9 can play a required pro-viral regulatory role in the virus life cycle of MYXV in monocytic cell lines when PKR is absent or repressed.

It is well known that certain individual virus-encoded immunomodulators can inhibit multiple ligands or pathways. For example, the M-T7 protein of MYXV binds and inhibits both rabbit interferon-gamma and diverse chemokines, the SECRET family of orthopoxvirus proteins can bind and co-inhibit both TNF and chemokines, the M-T5 intracellular host range factor of MYXV targets both Akt and the Skp1 component of the cellular SCF complex, and the M13 protein of MYXV targets both ASC-1 of the inflammasome complex and NF-κB1 [57], [58], [59], [60]. However, this is the first report of a single viral host range protein, M029, with two different functional cellular targets, one of which (PKR) is bound and inhibited to alleviate anti-viral signaling, while the other (RHA/DHX9) is bound and conscripted as a pro-viral effector to upregulate viral replication in a cell-specific fashion.

## Materials and Methods

### Ethics statement

All animal studies were performed following the IACUC protocol number IACUC #201005008 and titled “Studies in Poxvirus Host Range Genes and Tropism”. This protocol was approved by the University of Florida Animal Care Services in accordance with the guidelines set by the Association for Assessment and Accreditation of Laboratory Animal Care (AAALAC).

### Reagents and antibodies

Rabbit polyclonal antibody for PKR was purchased from Cell Signaling Technology. Rabbit polyclonal antibody for DHX9, mouse monoclonal antibody for DHX9 and PKR from Santa Cruz Biotechnology, rabbit polyclonal antibody for phospho PKR from Millipore, mouse monoclonal antibody for β-actin was obtained from Ambion and mouse antibody for V5 from Invitrogen. HRP-conjugated goat anti-rabbit and anti-mouse IgG antibodies were purchased from Jackson ImmunoResearch Laboratories. Generation of rabbit polyclonal and mouse monoclonal antibodies against MYXV proteins M-T7 and Serp1 was described before [61], [62].

### Cell lines and cell culture

Rabbit cell line RK13 (ATCC# CCL-37), human cell lines HeLa (ATCC# CCL-2), and A549 (ATCC# CCL-185), primary human fibroblasts GM02504 [40], monkey cell line BSC-40 (ATCC# CRL-2761), hamster kidney cell lines BHK21 (ATCC# CCL-10), mouse embryonic fibroblast cell lines NIH3T3 (ATCC# CRL-1658) all were cultured in Dulbecco minimum essential medium (DMEM; Invitrogen) supplemented with 10% fetal bovine serum (FBS; Gibco), 2 mM glutamine (Invitrogen) and 100 µg of penicillin-streptomycin (pen/strep; Invitrogen)/ml. Human monocytic THP1 (ATCC# TIB-202) and rabbit CD4+ T cells RL5 (Liu et al., 2011) were cultured in RPMI 1640 medium (Lonza, BioWhittaker) supplemented with 10% FBS, and 100 µg of pen/Sterp per ml. All cultures were maintained at 37°C in a humidified 5% CO_2_ incubator.

RK13 cells expressing VACV E3 protein (RK13-E3) was generated by transfecting RK13 cells with pcDNA3.1 (Geneticin resistance)-VACV E3L plasmid. Stably transfected cells were selected by the presence of 500 µg/ml Geneticin. Several clones were isolated and analyzed for expression of E3. The clone which showed highest level of E3 expression was used for virus propagation. RK13 cells expressing VACV E3 and K3 proteins (RK13-E3K3) was generated by transfecting RK13-E3 cells with pcDNA3.1 (Zeocin resistance)-VACV K3L-2×Flag tag plamid. Stably transfected cells were selected by the presence of 300 µg/ml Zeocin and 500 µg/ml Geneticin and maintained for 20 days under selection. The RK13-E3K3 cells were polyclonal.

### Construction of recombinant viruses

To make M029 mutant viruses, recombinant plasmids were first constructed using MultiSite Gateway Pro (Invitrogen) system. In one of the construct, vMyx-M029ID, an eGFP expression cassette (driven by a poxvirus synthetic early/late promoter [27]) was inserted into the M029 ORF. In another construct, vMyx-M029KO, the entire M029 gene was replaced by the eGFP expression cassette. For both the constructs upstream and downstream hybridizing sequences were amplified by PCR using specific primers to create entry clones by Gateway BP recombination system with appropriate pDONR vectors. The final recombination plasmids were constructed by recombination of three entry clones with a destination vector (pDEST40; Invitrogen) and using Gateway LR recombination system. The vMyx-M029ID and vMyx-M029KO viruses were constructed by infecting RK13-E3 and RK13-E3K3 cell lines respectively with wild-type MYXV Lausanne strain (vMyx-Lau), followed by transfection of the recombination plasmids. Multiple rounds of foci purifications were performed on the same cell lines based on eGFP expression and continued until pure foci of M029 mutant viruses were isolated. Virus was amplified from pure isolated foci and recombination was confirmed by PCR using appropriate primers (Figure 1). The recombinant virus, vMyx-M029V5N, where a N-terminal V5 tag was inserted in front of the M029 ORF was constructed using similar method. The virus also contains an eGFP expression cassette driven by a poxvirus synthetic early/late promoter in the M135-136 locus [28]. The revertant myxoma viruses for M029 mutants, vMyx-M029ID-Rev and vMyx-M029KO-Rev were constructed by infecting nonpermissive (for M029 mutants) BSC40 cells with M029 mutants, followed by transfection with a revertant plasmid containing the myxoma virus M028, M029 and M030 gene sequences. The viruses were purified by multiple rounds of focus purification based on nonfluorescent foci formation, which was confirmed by PCR using the appropriate primers. In these revertant viruses an eGFP cassette was also inserted in the M135-136 locus.

### Single-step growth curve

Cells were seeded into twelve-well dishes and semi-confluent monolayers (1×10^5^ cells) were infected with different myxoma viruses at an MOI of 5 for one hour. The virus containing media was removed, and the cell monolayer was washed with complete medium and incubated with fresh complete medium. Samples were collected at various times post-infection and stored in −80°C until processed. The samples were freeze-thawed at −80°C and 37°C for three times and sonicated for one minute to release the viruses from infected cells. The virus was titrated back onto RK13-E3L monolayers by serial dilution in triplicate. After 48 hrs of infection the numbers of fluorescent foci were counted from each dilution and calculated the virus titer.

### Viral preparation

Construction of a wild-type MYXV that express GFP under the control of a synthetic VACV early-late promoter was described previously [28]. The vMyx-M029KO and vMyx-M029ID viruses were grown and amplified in RK13-E3 cells. All other myxoma viruses were grown in BSC40 cells. Viruses were purified by centrifugation through a sucrose cushion and two successive sucrose gradient sedimentations as described previously [60]. Vesicular stomatitis virus (VSV) expressing GFP was prepared as described before [63].

### Western blot analysis

The mock or infected cells were harvested at different time points after infection with viruses, washed with PBS and stored at −80°C or processed immediately with RIPA lysis buffer (50 mMTris, 150 mMNaCl, 0.1% SDS, 0.5% sodium deoxycholate, 1% NP40, 1 mM PMSF, and protease inhibitor cocktail (Roche). Amount of total proteins were estimated by Bradford assay (Bio-Rad) and equal amount of total proteins were used for Western blot analysis as described before [64]. Briefly, protein samples were separated on 10% SDS-PAGE gels and transferred to PVDF membrane (GE Healthcare) using a wet transfer apparatus (Invitrogen). Membranes were blocked in TBST buffer (20 mM Tris, 150 mM NaCl, 0.1% Tween-20 pH 7.6) containing 5% non-fat dry milk for 1 hr at room temperature and then incubated overnight with appropriate primary antibody at 4°C. The membranes were washed three times, 15 minutes each with TBST and incubated with HRP-conjugated goat-anti-mouse (1∶10,000) or goat anti-rabbit (1∶10,000) secondary antibody in TBST containing 5% non-fat dry milk for 1 hour at room temperature with gentle agitation and were then washed three times, 15 minutes each with TBST. The proteins were detected using the chemiluminescence substrate (Pierce) and exposure to X-ray film (Kodak). Protein bands on X-ray films were quantified by using ImageJ software (rsb.info.nih.gov/ij).

### Transfection of siRNAs and construction of stable cell lines using lentivirus packaged with short hairpin RNAs (shRNAs)

For siRNA transfection, cells were seeded at 5×10^4^ cells per well in 24-well plates in growth medium without antibiotics. PKR (target sequences: GUAAGGGAACUUUGCGAUA; GCGAGAAACUAGACAAAGU; CGACCUAACACAUCUGAAA; CCACAUGAUAGGAGGUUUA) and DHX9 (target sequences: GGAUUAAACUGCAAAUAUC; GGCUUUGGUUGUUGAAGUA; CAAACAACCUGCUAUCAUC; GUAAAUGAACGUAUGCUGA) siRNAs used were ON-TARGET*plus* SMART pool siRNA purchased from Thermo Scientific (Dharmacon). For transfection, siRNA solution (50–100 nM final concentration/well) was prepared in 50 µl Opti-MEM I Reduced serum medium (Invitrogen). Lipofectamine RNAiMAX (Invitrogen) solution was prepared in 50 µl Opti-MEM I Reduced serum medium and incubated 5 min at room temperature. siRNA and lipofectamine solutions were mixed and incubated for 20 min at room temperature and added to the cells to make final volume of 500 µl and incubated at 37°C in a CO_2_ incubator. The knock down was verified after 48–72 h of transfection. For infection, the cells were infected with viruses after 48 h of post-transfection.

Lentivirus particles packaged with human PKR shRNAs or control shRNAs were purchased from Snata Cruz Biotechnology Inc. They were used independently to infect cells to construct stable cell lines according to the manufacturer's protocol. After selection of puromycin (Sigma) antibiotic resistant cells, Western blot analysis was performed to determine the level of PKR knockdown.

### Coimmunoprecipitation (co-IP) and mass spectrometry of protein samples

For co-IP, cells were lysed using RIPA lysis buffer. The cleared cell lysates after centrifugation for 15 min at 12,000 rpm at 4°C were incubated with Pierce protein A/G agarose (Thermo scientific) for pre-clearing. The agarose beads were removed by centrifugation for 15 min at 12,000 rpm at 4°C. The supernatants were incubated with a monoclonal anti-V5 antibody (Invitrogen) for 1 h at 4°C and followed by incubation with protein A/G agarose for over-night at 4°C. In some cases cell lysates were treated with RNase A/T1 (Fermentas) or RNase V1 (Invitrogen) overnight at 4°C. The agarose beads were pelleted by centrifugation at 2,000 rpm for 2 minutes, washed four times with lysis buffer and samples were then analyzed by Western blotting.

For mass spectrometry, protein samples from co-IP were separated on a 12% SDS-PAGE gel by electrophoresis and stained with mass spectrometry-compatible Coomassie blue (SimplyBlue SafeStain; Invitrogen) according to the manufacturer's protocol. Unique protein bands present in the vMyxM029V5N virus infected samples compared to the controls were dissected for trypsin digestion, followed by liquid chromatography-tandem mass spectrometry analysis (done at the ICBR, UF, proteomic core). The results were searched against protein database and then analyzed and displayed with matching polypeptide sequences in the identified protein sequence using Scaffold software (Proteome Software, Portland, OR).

### Immunofluorescence microscopy

RK-13 cells grown on glass coverslips, were mock-infected or infected with vMyxGFP or vMyxM029V5N for 3 h or 24 h at an MOI of 5.0 or 0.1, respectively. After infection, cells were fixed in 4% paraformaldehyde in PBS for 10 min at room temperature and permeabilized in 0.1% Triton X-100 in PBS for 10 min. Cells were quenched in 0.1 M Glycine in PBS for 20 min and then blocked in 1% BSA in PBS for 30 min. Cells were incubated with an anti-V5 monoclonal antibody, followed by Texas Red-conjugated goat anti-mouse antibody (Jackson ImmunoResearch Laboratories). DNA in the nuclei and viral factory was stained with 4′,6-diamidino-2-phenylindole (DAPI). Images were captured on a Leica laser scanning confocal microscope.

### RNA purification and real-time PCR

Total RNA was purified from 10^6^ cells plated in each well of a six-well dish. The following day cells were infected with viruses. At the given time points, cells were lysed with RLT buffer (Qiagen), and total RNA was extracted using RNeasy kit (Qiagen). Generally, 1–2 µg of total RNA was used to make cDNA. Genomic DNA was removed from total RNA using DNA-free kit (Ambion) according to the manufacturer's recommendations. Following the removal of genomic DNA, cDNA was prepared using Superscript III reverse transcriptase (Invitrogen) [65]. PCR amplification was performed for 35 cycles using the following cycling conditions: 94° for 1 min, followed by 35 cycles of 94° for 30 seconds, 56° for 30 seconds, 72° for 1 min, and then a final extension of 72° for 10 min. Forward and reverse primer pairs are listed in supplementary Table 1. Lack of DNA contamination in the RNA preparation was verified by PCR amplification in the absence of reverse transcription.

### Animal studies

New Zealand White rabbits were purchased from Charles River Laboratories International. The animal study was approved by the Institutional Animal Care and Usage Committee (IACUC) at the University of Florida and studies were performed as described before [66]. For virus injection, 1000 focus-forming units (FFU) of the tested virus was resuspended in 100 µl of PBS and inoculated intradermally in the left flank of each rabbit. Daily physical examinations were conducted to evaluate rabbits condition by monitoring respiration, weight, temperature, heart rate, lung sound, food and water intake, urine and feces output, hydration status, attitude, posture and indications of primary lesion and appearance of secondary lesions. Based on the evaluations, the rabbits received daily clinical score that ranged from 0 to 34 (the maximum score). The animals were humanely euthanized when the clinical score reached 26 to 34, had open mouth breathing due to respiratory stress, orthopnea, cyanosis or no food and water intake for 48 h. The animals that survived challenge with M029 mutant virus infection, were re-challenged with a lethal dose of vMyx-Lau (1000 FFU) by the intradermal route in the right flank of the animal.

### Statistics

Data were expressed as means ± SD and were analyzed by paired *t*-test. Significant difference was accepted at *p*<0.05.

## Supporting Information

Figure S1 **MYXV M028 and M030 gene transcripts can be detected from M029-defective virus infection.** Total RNA was extracted from vMyx-GFP, vMyx-M029ID and vMyx-M029KO virus-infected RK13 cells after 24 h p.i. and subjected to RT-PCR using specific primers for M028, M029, M030 and rabbit GAPDH (as control). The amplified products were resolved on a 1.5% agarose gel and the bands were visualized by SYBR Green I nucleic acid gel stain (Invitrogen).
(TIF)

Figure S2 **M029 protein interacts with human PKR via dsRNA in virus-infected cells.** HeLa cells were infected with vMyx-M029V5N for 24 h, cell lysates were treated with RNase V1 (10 u/ml) or RNase A/T1 at 4°C over-night and co-IP was performed using mouse anti-V5 antibody. After Western transfer the blot was probed with the indicated antibodies.
(TIF)

Figure S3 **PKR protein interacts directly with DHX9 in HeLa cells independent of dsRNA.** Uninfected HeLa cell lysates were treated with RNase V1 (10 u/ml) at 4°C over-night and co-IP was performed using rabbit anti-DHX9 antibody. After Western transfer the blot was probed with mouse anti-PKR and anti-DHX9 antibodies.
(TIF)

Figure S4 **MYXV early and late gene transcripts remain unchanged in the absence of DHX9.** THP1, shPKR-THP1 and shPKR-THP1 cells transfected with DHX9 siRNA were infected with vMyx-GFP and vMyx-M029ID viruses for indicated time points without (3 and 24) or with araC (24a). Total RNA was extracted from these cells and subjected to RT-PCR using specific primers for M-T7 (early gene), Serp-1 (late gene) and human GAPDH (as control). The amplified products were resolved on a 1.5% agarose gel and the bands were visualized by SYBR Green I nucleic acid gel stain.
(TIF)

Figure S5 **Rabbit type I IFN restricts the replication of M029-defective MYXV in rabbit cells.** RK13 cells were transfected with poly I∶C (InvivoGen) overnight and the induced supernatants were harvested for assay. A) RK13 cells were infected with the indicated viruses with an MOI of 0.1 in the absence or presence of the rabbit IFN containing supernatant. Fluorescence images were taken after 48 h p.i. B) Virus was tittered from the infected cell lysates after 48 h p.i. using RK13-E3 cells. * *P*<0.05 compared with untreated samples.
(TIF)

Table S1 **Primers for RT-PCR.**
(DOCX)
