# Supplementary figures and images for: Myxoma Virus Protein M029 Is a Dual Function Immunomodulator that Inhibits PKR and Also Conscripts RHA/DHX9 to Promote Expanded Host Tropism and Viral Replication
Source: PLoS Pathog. 2013 Jul 4;9(7):e1003465. doi: 10.1371/journal.ppat.1003465 (PMC3701710; doi:10.1371/journal.ppat.1003465)

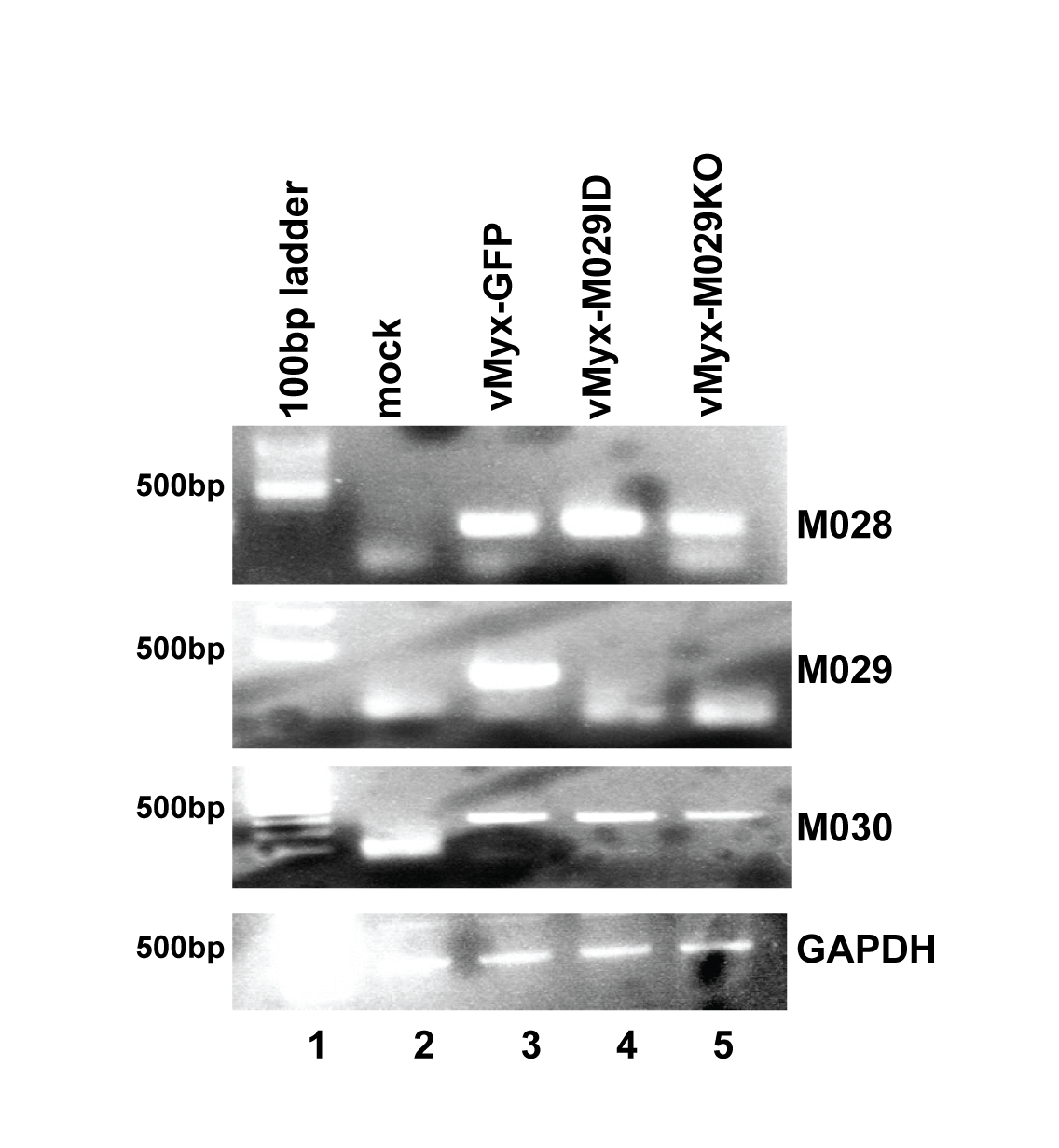

Supplement: Figure S1 — MYXV M028 and M030 gene transcripts can be detected from M029-defective virus infection. Total RNA was extracted from vMyx-GFP, vMyx-M029ID and vMyx-M029KO virus-infected RK13 cells after 24 h p.i. and subjected to RT-PCR using specific primers for M028, M029, M030 and rabbit GAPDH (as control). The amplified products were resolved on a 1.5% agarose gel and the bands were visualized by SYBR Green I nucleic acid gel stain (Invitrogen). (TIF) [file ppat.1003465.s001.tif]

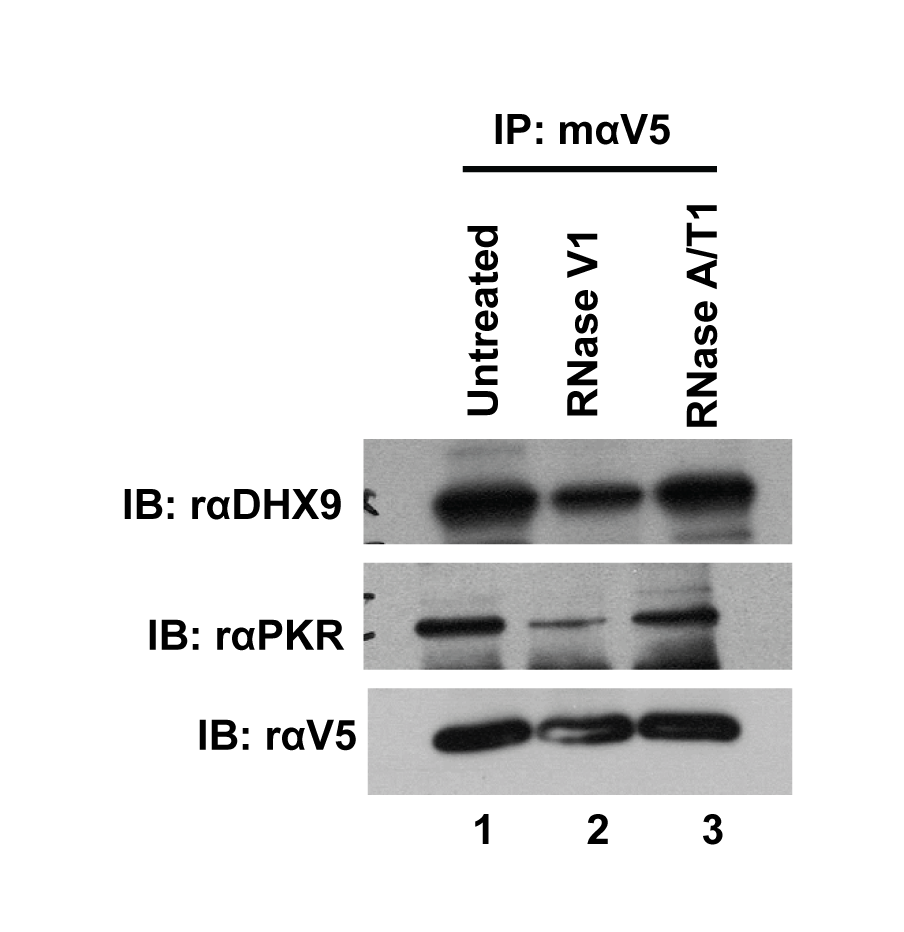

Supplement: Figure S2 — M029 protein interacts with human PKR via dsRNA in virus-infected cells. HeLa cells were infected with vMyx-M029V5N for 24 h, cell lysates were treated with RNase V1 (10 u/ml) or RNase A/T1 at 4°C over-night and co-IP was performed using mouse anti-V5 antibody. After Western transfer the blot was probed with the indicated antibodies. (TIF) [file ppat.1003465.s002.tif]

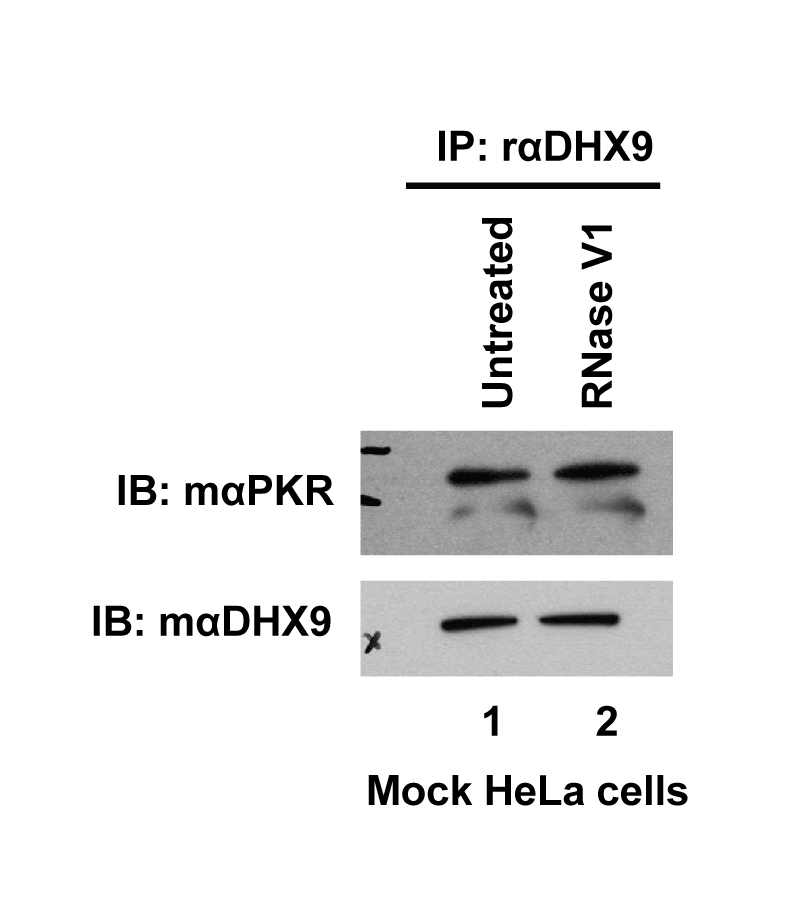

Supplement: Figure S3 — PKR protein interacts directly with DHX9 in HeLa cells independent of dsRNA. Uninfected HeLa cell lysates were treated with RNase V1 (10 u/ml) at 4°C over-night and co-IP was performed using rabbit anti-DHX9 antibody. After Western transfer the blot was probed with mouse anti-PKR and anti-DHX9 antibodies. (TIF) [file ppat.1003465.s003.tif]

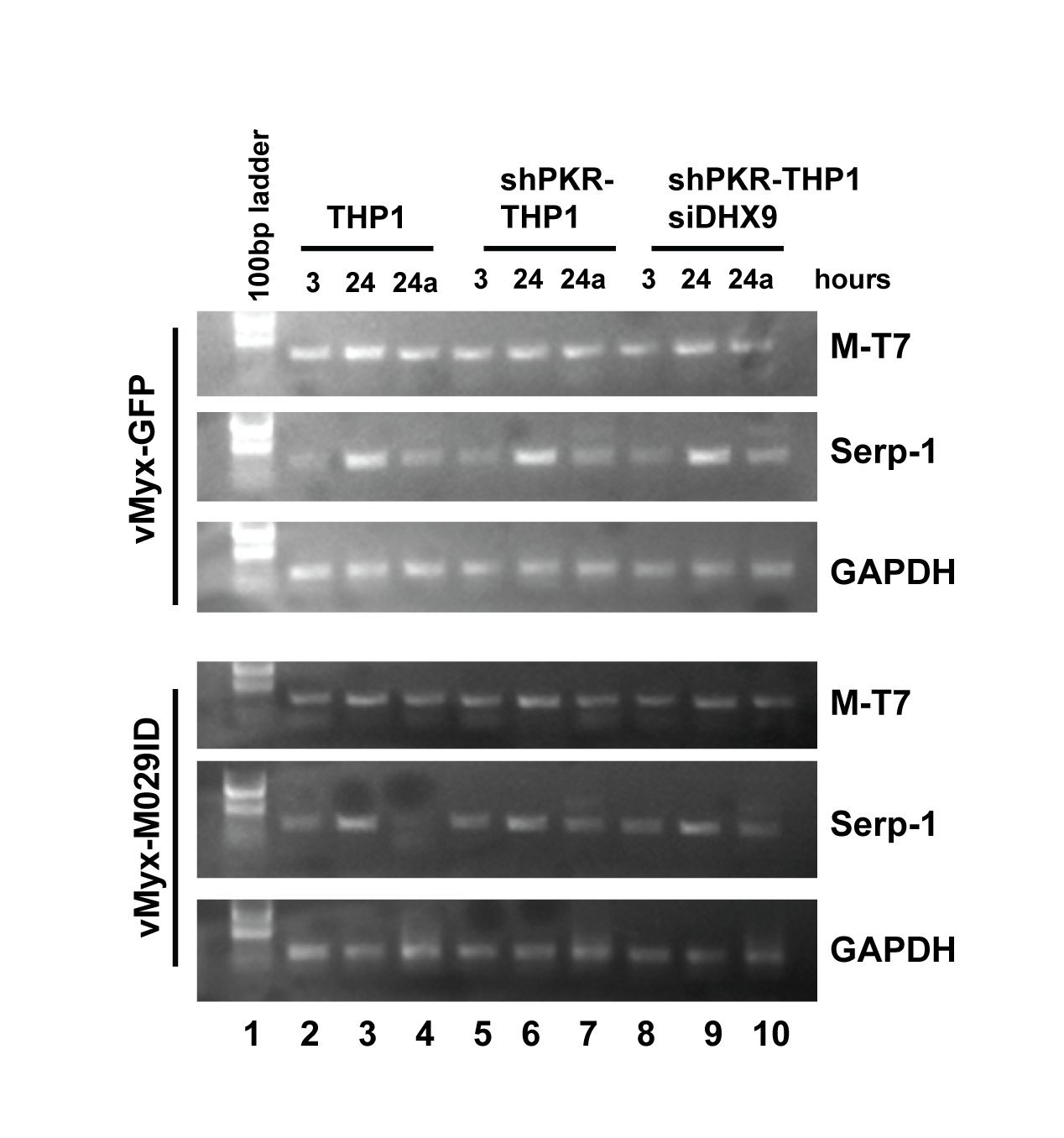

Supplement: Figure S4 — MYXV early and late gene transcripts remain unchanged in the absence of DHX9. THP1, shPKR-THP1 and shPKR-THP1 cells transfected with DHX9 siRNA were infected with vMyx-GFP and vMyx-M029ID viruses for indicated time points without (3 and 24) or with araC (24a). Total RNA was extracted from these cells and subjected to RT-PCR using specific primers for M-T7 (early gene), Serp-1 (late gene) and human GAPDH (as control). The amplified products were resolved on a 1.5% agarose gel and the bands were visualized by SYBR Green I nucleic acid gel stain. (TIF) [file ppat.1003465.s004.tif]

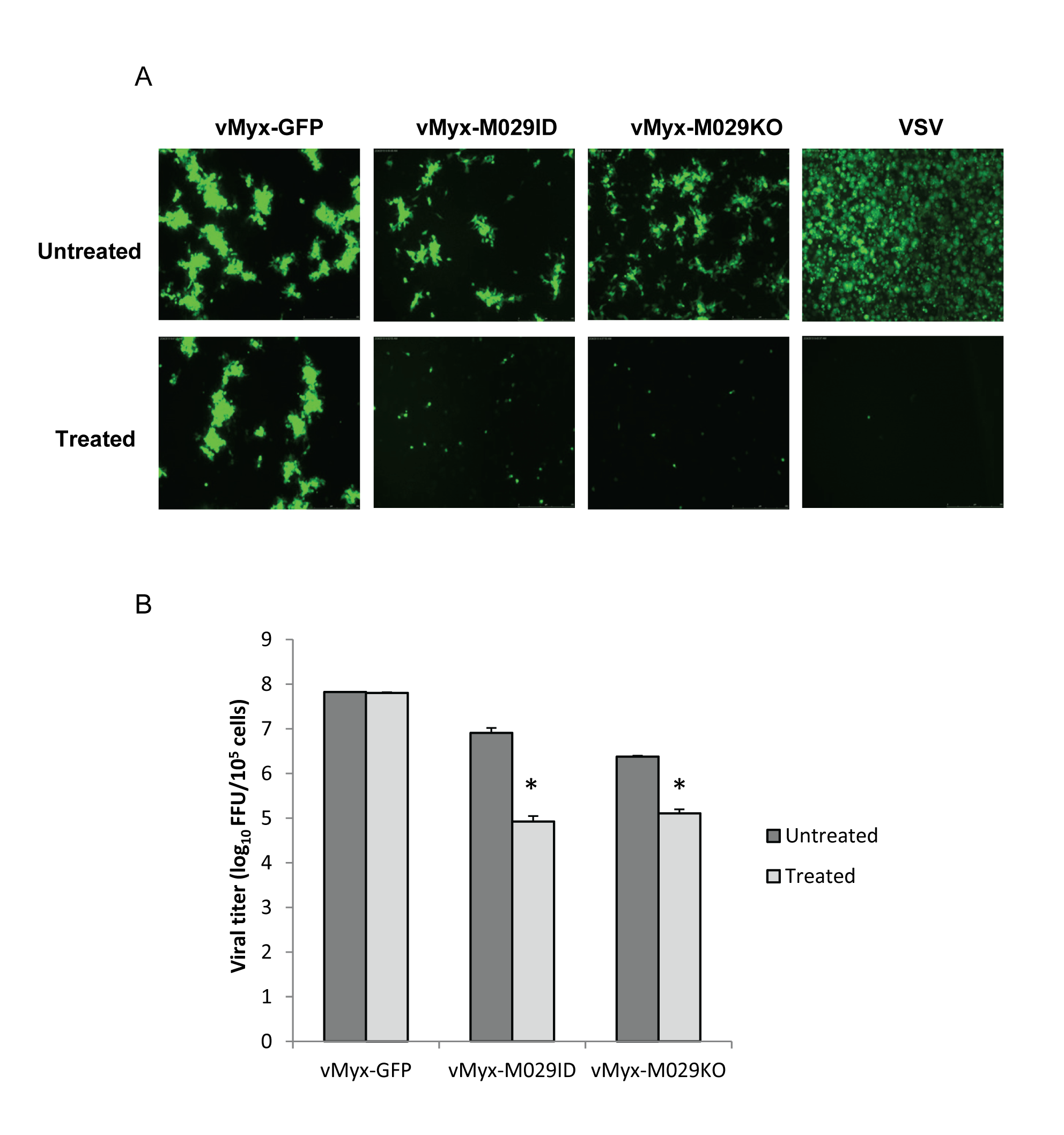

Supplement: Figure S5 — Rabbit type I IFN restricts the replication of M029-defective MYXV in rabbit cells. RK13 cells were transfected with poly I∶C (InvivoGen) overnight and the induced supernatants were harvested for assay. A) RK13 cells were infected with the indicated viruses with an MOI of 0.1 in the absence or presence of the rabbit IFN containing supernatant. Fluorescence images were taken after 48 h p.i. B) Virus was tittered from the infected cell lysates after 48 h p.i. using RK13-E3 cells. * P<0.05 compared with untreated samples. (TIF) [file ppat.1003465.s005.tif]
