# Supplementary material for: Myxoma Virus Protein M029 Is a Dual Function Immunomodulator that Inhibits PKR and Also Conscripts RHA/DHX9 to Promote Expanded Host Tropism and Viral Replication
Source: PLoS Pathog. 2013 Jul 4;9(7):e1003465. doi: 10.1371/journal.ppat.1003465 (PMC3701710; doi:10.1371/journal.ppat.1003465)
Supplement: Table S1 — Primers for RT-PCR. (DOCX) [file ppat.1003465.s006.docx]

**Table S1: Primers for RT-PCR**

| **Gene** | **Forward primer sequence (5’-3’)** | **Reverse primer sequence (5’-3’)** |
| --- | --- | --- |
| M028 | GTTACCAAATGCGTGCGGTT | ATACCGTCGTTGTTGGGGAC |
| M029 | ATGGATCCCATTAACACGCT | TTAAAACTTTATAACGACGTGTT |
| M030 | ACCGTCTCCCAGAACGAGTA | TGCAGATGTCCTCGTATCGC |
| M-T7 | ATGTGTGTGAATGGGCGTCT | CGACGTACTCCGCTCGTTTA |
| Serp-1 | TGCTGCTATTGAGCTCCGTT | TGTTGCGTATCCAGAAGCGT |
| Rabbit GAPDH | CAAGCCTCTAGCCCACGTA | GGCAATGATCCCAAAGTAG |
| Human GAPDH | GAAGGTGAAGGTCGGAGTC | GAAGATGGTGATGGGATTTC |
